# Engineering extracellular vesicles for inflammatory bowel disease and colitis-associated colorectal cancer: from therapeutic innovation to clinical translation

**DOI:** 10.3389/fimmu.2026.1933602

**Published:** 2026-10-07

**Authors:** Minrui Huang, Jintao Yuan, Peter Muro, Tao Jin, Fei Mao, Wenjun Zhu

**Affiliations:** 1 Department of Laboratory Medicine, School of Clinical Medicine and Laboratory Medicine, Jiangsu University, Zhenjiang, Jiangsu, China; 2 The People’s Hospital of Danyang, Affiliated Danyang Hospital of Nantong University, Zhenjiang, Jiangsu, China; 3 Department of Gastrointestinal and Endoscopy, The Affiliated Yixing Hospital of Jiangsu University, Wuxi, Jiangsu, China; 4 Department of Laboratory Medicine, The Second People's Hospital of Lianyungang, Lianyungang, China; 5 Department of Laboratory Medicine, Affiliated Lianyungang Clinical College of Jiangsu University, Lianyungang, China

**Keywords:** colitis-associated colorectal cancer, extracellular vesicle engineering, extracellular vesicles, IBD, targeted drug delivery

## Abstract

Inflammatory bowel disease (IBD), encompassing Crohn’s disease and ulcerative colitis, is a chronic relapsing inflammatory disorder that increases the risk of colitis-associated colorectal cancer (CAC). Despite major advances in biologics and small-molecule therapies, many patients experience primary or secondary treatment failure, adverse effects, and disease recurrence, underscoring the need for more effective, targeted therapeutic strategies. Extracellular vesicles (EVs), including exosomes, microvesicles, and apoptotic bodies, have emerged as promising nanoscale therapeutic platforms owing to their intrinsic biocompatibility, low immunogenicity, cargo-protective lipid bilayer, and natural ability to mediate intercellular communication. Recent advances in EV engineering have further expanded their therapeutic potential through genetic modification of parental cells, cargo loading, surface functionalization, and hybrid biomaterial systems, enabling enhanced targeting, drug delivery, immune modulation, tissue regeneration, and anti-tumor activity. This review summarizes the biological characteristics of EVs and discusses recent progress in engineering strategies aimed at improving their therapeutic efficacy in IBD and CAC. We critically evaluate current preclinical evidence regarding immunomodulation, restoration of intestinal barrier integrity, regulation of gut microbiota, targeted drug delivery, cancer immunotherapy, and inhibition of metastasis. Finally, we discuss the major translational challenges, including manufacturing standardization, quality control, large-scale production, safety evaluation, regulatory considerations, and future perspectives necessary for successful clinical implementation of engineered EV-based therapies.

## 1 Introduction

Inflammatory bowel disease (IBD) is a chronic, idiopathic, relapsing inflammatory disorder of the gastrointestinal tract that primarily comprises two major subtypes: ulcerative colitis (UC) and Crohn’s disease (CD). UC is characterized by continuous mucosal inflammation confined to the colon and rectum, whereas CD can affect any segment of the gastrointestinal tract and is characterized by transmural inflammation and discontinuous lesions (1, 2). Although the precise etiology of IBD remains incompletely understood and no curative therapy is currently available, the disease is widely recognized as arising from complex interactions among genetic susceptibility, environmental factors, gut microbial dysbiosis, epithelial barrier dysfunction, and dysregulated immune responses (3, 4). These interactions result in persistent intestinal inflammation and progressive tissue injury that contribute to disease chronicity. IBD is a systemic disease that extends beyond the gastrointestinal tract. Patients commonly present with abdominal pain, diarrhea, rectal bleeding, and weight loss, while approximately half also develop extraintestinal manifestations (EIMs), most frequently involving the joints, skin, and eyes, although the liver, pancreas, and other organs may also be affected (5, 6). Peripheral arthritis, episcleritis, and erythema nodosum generally parallel intestinal disease activity and often improve following treatment of intestinal inflammation, whereas anterior uveitis, ankylosing spondylitis, and primary sclerosing cholangitis frequently occur independently of intestinal disease activity (6). Consequently, IBD substantially impairs quality of life and imposes an increasing healthcare and socioeconomic burden worldwide.

Current treatment strategies for IBD primarily include aminosalicylates, corticosteroids, immunomodulators, biologics, and, more recently, small-molecule agents, with surgery reserved for patients with refractory disease or complications (7). These therapies have improved disease management by inducing and maintaining remission; however, several important challenges remain. Treatment efficacy is frequently limited by adverse effects, primary non-response, secondary loss of response during long-term therapy, and the inability to achieve sustained mucosal healing in many patients (8). Furthermore, existing therapies largely focus on suppressing inflammation rather than restoring intestinal barrier function, correcting gut microbial dysbiosis, or preventing long-term complications such as fibrosis and colorectal cancer. Therefore, there remains an urgent need to develop novel therapeutic approaches that provide targeted, durable, and disease-modifying effects while minimizing systemic toxicity.

Colorectal cancer (CRC) is the third most frequently diagnosed malignancy and the second leading cause of cancer-related mortality worldwide, with both incidence and mortality continuing to increase (9). Patients with longstanding IBD are at significantly greater risk of developing colitis-associated colorectal cancer (CAC), a distinct subtype of CRC driven by chronic intestinal inflammation. Several retrospective studies and meta-analyses have demonstrated that patients with UC have approximately a 1.7-fold increased risk of CRC compared with the general population (10). Similarly, patients with CD exhibit an elevated CRC risk, with pooled standardized incidence ratios (SIRs) of 1.9 (95% CI, 1.4–2.5) for CRC and 2.5 (95% CI, 1.7–3.5) for colon cancer (11), while the pooled SIR for all patients with IBD is approximately 1.7 (95% CI, 1.2–2.2) (12). Unlike sporadic CRC, CAC typically develops through an inflammation–dysplasia–carcinoma sequence driven by persistent inflammatory injury (13). An increased inflammatory burden is strongly associated with CRC development, with each 10-unit increase in the inflammatory index corresponding to an estimated 2.1% increase in CRC risk (14). Chronic inflammation promotes carcinogenesis through oxidative stress, sustained activation of pro-inflammatory cytokines including tumor necrosis factor (TNF), interleukin-1 (IL-1), interleukin-6 (IL-6), interleukin-17A (IL-17A), and interleukin-23 (IL-23), and persistent activation of the nuclear factor-kappa-B(NF-κB) and signal transducer and activator of transcription 3 (STAT3) signaling pathways (15, 16). Excessive production of reactive oxygen species (ROS) and reactive nitrogen species (RNS) overwhelms cellular antioxidant defenses, resulting in oxidative damage to lipids, proteins, and DNA, including 8-oxo-guanine formation, base substitutions, and single- and double-strand DNA breaks (17–19). Together with immune cell-derived inflammatory mediators and gut microbiota-derived signals, these molecular alterations promote activation of oncogenes, inactivation of tumor suppressor genes, genomic instability, and malignant transformation (13, 20). The molecular evolution of CAC also differs from that of sporadic CRC. In CAC, tumor protein p53 (TP53) mutations frequently occur at an early stage, often preceding dysplasia, whereas antigen-presenting cell (APC) mutations generally arise later and less frequently (13). Moreover, the risk of CRC increases with disease duration, extensive colonic involvement, persistent inflammatory activity, and concomitant primary sclerosing cholangitis.

In contrast, effective anti-inflammatory treatment with agents such as 5-aminosalicylic acid, mesalazine, and corticosteroids has been associated with a reduced risk of CRC (21–23). Gut microbial dysbiosis further contributes to inflammation-associated carcinogenesis. IBD-associated dysbiosis is characterized by depletion of beneficial commensal bacteria, expansion of opportunistic pathogens, and reduced microbial diversity (24, 25). This microbial imbalance disrupts epithelial barrier integrity, perpetuates chronic inflammation, and promotes carcinogenesis through the production of harmful metabolites, including trimethylamine N-oxide, secondary bile acids, hydrogen sulfide, and N-nitroso compounds (26). Collectively, these findings highlight the need for therapeutic strategies that simultaneously suppress chronic inflammation, restore intestinal homeostasis, and prevent inflammation-driven colorectal carcinogenesis. Extracellular vesicles (EVs) are naturally occurring lipid bilayer-enclosed nanoparticles that include exosomes, microvesicles, and apoptotic bodies and function as important mediators of intercellular communication by transporting proteins, lipids, messenger RNAs, microRNAs, and other bioactive molecules (27–31). Owing to their excellent biocompatibility, low immunogenicity, intrinsic targeting capability, and ability to protect therapeutic cargo from degradation, EVs have emerged as highly promising drug delivery vehicles and therapeutic agents (32). Recent advances in EV engineering have further expanded their therapeutic potential by enabling precise cargo loading, surface modification, and targeted delivery of therapeutic molecules. Engineered EVs can effectively modulate immune responses, restore intestinal barrier integrity, regulate gut microbiota, and inhibit tumor progression while minimizing systemic toxicity (29, 30, 33). These unique biological properties position engineered EVs as attractive next-generation therapeutic platforms capable of addressing both chronic intestinal inflammation and colitis-associated colorectal cancer.

Unlike previous reviews that have primarily focused on the biological roles or therapeutic applications of EVs in IBD or CRC separately, this review specifically examines engineered EVs across the IBD–CAC continuum. Rather than considering intestinal inflammation and malignant transformation as distinct therapeutic settings, we integrate EV design, including donor cell selection, cargo loading, surface modification, and delivery strategies, with stage-specific pathological barriers, such as immune dysregulation, epithelial barrier disruption, gut dysbiosis, persistent inflammation, and the tumor microenvironment. This integrated framework is particularly relevant because CAC represents an inflammation-driven malignancy that develops through an inflammation–dysplasia–carcinoma sequence. Accordingly, EV platforms designed to restore intestinal homeostasis may offer opportunities for cancer prevention or interception, whereas EVs targeting established CAC may require additional tumor-targeting and immunomodulatory functions. By linking these preventive and therapeutic objectives, this review aims to identify rational engineering strategies for developing precision EV-based interventions across the progression from IBD to CAC.

This review summarizes recent advances in extracellular vesicle engineering for the treatment of inflammatory bowel disease and colitis-associated colorectal cancer. We first discuss the biological characteristics of EVs and the major engineering strategies developed to improve their targeting efficiency and therapeutic performance. We then critically review the current evidence supporting the use of engineered EVs for immune modulation, intestinal barrier repair, gut microbiota regulation, targeted drug delivery, cancer immunotherapy, and the inhibition of angiogenesis and metastasis. Finally, we discuss the major translational challenges, including large-scale manufacturing, standardization, safety, and regulatory considerations, as well as future perspectives on the clinical translation of engineered EV-based therapies.

## 2 Biology of extracellular vesicles and their therapeutic potential

### 2.1 Definition and classification of extracellular vesicles

EVs are membrane-bound nanoparticles secreted by virtually all cell types into the extracellular environment. They serve as important mediators of intercellular communication by transporting a wide range of biomacromolecules, including proteins, lipids, and nucleic acids, between neighboring and distant cells (27). EVs are widely distributed in biological fluids and tissues and participate in numerous physiological and pathological processes. Based on their biogenesis, size, and biological characteristics, EVs are generally classified into three major subtypes: exosomes, microvesicles (MVs), and apoptotic bodies (27). Exosomes are the smallest EV subtype, typically measuring 30–150 nm in diameter. They originate from the endosomal pathway and are released into the extracellular space following fusion of multivesicular bodies with the plasma membrane (34). EVs have been identified in numerous biological fluids, including blood, urine, saliva, breast milk, amniotic fluid, synovial fluid, ascitic fluid, and adipose tissue (35). Owing to their small size, EVs readily diffuse through tissues, enter the circulation, and are capable of crossing biological barriers such as the blood-brain barrier (BBB) (36). Their remarkable stability and ability to transfer bioactive cargo enable EVs to regulate diverse physiological and pathological processes in recipient cells, making them promising candidates for targeted drug delivery (36, 37). Microvesicles are generally larger than exosomes, ranging from approximately 100 to 1000 nm in diameter. Unlike exosomes, they are generated through direct outward budding of the plasma membrane and carry a diverse repertoire of proteins, lipids, and nucleic acids that reflect the physiological status of their parent cells (28). Apoptotic bodies are the largest EV subtype, measuring approximately 1–5 μm in diameter. They are produced during programmed cell death and typically contain fragmented DNA, RNA, proteins, and cytoplasmic organelles derived from apoptotic cells (38). Although exosomes, microvesicles, and apoptotic bodies differ in their origin, size, and biological properties, they all function as important mediators of intercellular communication by transporting bioactive molecules that regulate immune responses, tissue homeostasis, and disease progression (39).

### 2.2 Biogenesis and molecular composition of EVs

The biogenesis of EVs is a highly regulated process involving multiple intracellular pathways. Exosomes originate from the endosomal system through the formation of multivesicular bodies. In contrast, microvesicles are produced by outward budding of the plasma membrane, and apoptotic bodies are generated during cellular apoptosis (37, 40). Despite these distinct mechanisms, all EVs possess a phospholipid bilayer that is essential for maintaining vesicle stability and protecting their molecular cargo (39). The lipid bilayer not only shields encapsulated molecules from enzymatic degradation but also facilitates targeted interactions with recipient cells through membrane proteins and receptor-ligand recognition, thereby enhancing the efficiency of intercellular communication and cargo delivery (31, 41). EVs transport a diverse array of biologically active molecules, including proteins, lipids, messenger RNAs (mRNAs), microRNAs (miRNAs), long non-coding RNAs (lncRNAs), circular RNAs (circRNAs), and other regulatory nucleic acids (27, 42). Proteins contribute to membrane integrity, receptor recognition, intracellular signaling, and cargo transport (43), whereas lipids maintain membrane fluidity and regulate EV uptake by recipient cells (44). Among the nucleic acids, miRNAs have received particular attention because they regulate post-transcriptional gene expression by suppressing target mRNAs, thereby influencing numerous biological processes, including inflammation, immune regulation, tissue repair, and tumor progression (28, 45, 46). The diverse molecular composition of EVs underlies their functional versatility and provides the biological basis for their therapeutic applications.

### 2.3 Biological roles of endogenous EVs in IBD

IBD, including CD and UC, is characterized by chronic intestinal inflammation resulting from dysregulated interactions among immune cells, intestinal epithelial cells, and the gut microbiota (47). Growing evidence indicates that extracellular vesicles are important mediators of these interactions and contribute to both the pathogenesis and resolution of intestinal inflammation (48). Through the transfer of bioactive molecules, endogenous EVs regulate immune responses, maintain intestinal barrier integrity, and facilitate communication between the gut microbiota and the host, thereby influencing intestinal homeostasis (**Figure 1**).

**Figure 1:**
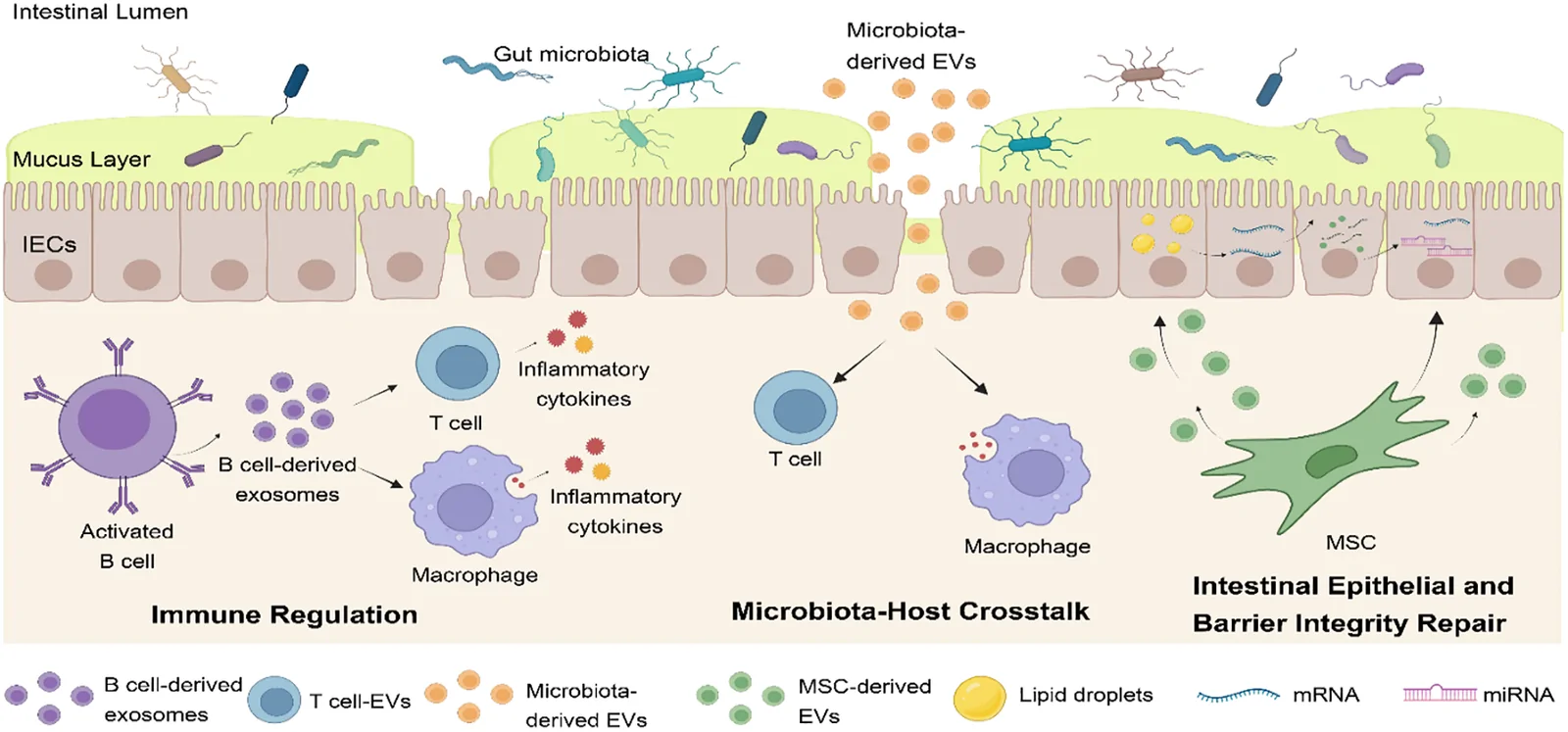
Biological functions of endogenous EVs in maintaining intestinal homeostasis and regulating IBD. Endogenous EVs derived from immune cells, gut microbiota, and MSCs mediate intercellular communication within the intestinal microenvironment. B cell-derived EVs regulate antigen presentation and T-cell activation, while immune cell-derived cytokines coordinate inflammatory responses. Microbiota-derived EVs facilitate bidirectional communication between the gut microbiota and the host, contributing to immune regulation and maintenance of intestinal homeostasis. In parallel, MSC-derived EVs deliver bioactive molecules, including lipids, mRNAs, and microRNAs, to intestinal IECs, promoting epithelial regeneration, restoring barrier integrity, and enhancing mucosal repair. Together, these coordinated EV-mediated mechanisms regulate immune homeostasis, microbiota-host crosstalk, and intestinal epithelial barrier function, providing the biological basis for the therapeutic application of engineered EVs in IBD and CRC.

#### 2.3.1 EV-mediated immune modulation

EVs regulate both innate and adaptive immune responses by mediating communication among immune cells. Studies have demonstrated that B-cell-derived EVs can present antigens, directly activate T cells, regulate T-cell function, and influence macrophage activity, thereby modulating intestinal inflammatory responses (49). Depending on their cellular origin and molecular cargo, EVs may exert either pro-inflammatory or anti-inflammatory effects, highlighting their central role in maintaining immune homeostasis during IBD (50).

#### 2.3.2 Regulation of intestinal epithelial barrier integrity

Maintenance of intestinal barrier integrity is essential for preventing excessive immune activation and microbial translocation. Mesenchymal stem cell (MSC)-derived EVs have been shown to mimic many of the reparative properties of their parent cells by delivering bioactive molecules, including proteins, lipids, mRNAs, and miRNAs, to intestinal epithelial cells. These cargoes promote epithelial cell proliferation, migration, and survival, thereby facilitating mucosal repair and strengthening epithelial barrier function in experimental models of IBD (51).

#### 2.3.3 Gut microbiota-host communication

Recent studies have identified microbiota-derived EVs as important mediators of communication between intestinal microorganisms and the host immune system. These vesicles help regulate microbial homeostasis, intestinal immune responses, and epithelial barrier function, thereby influencing both the initiation and progression of intestinal inflammation (52). Dysregulation of microbiota-derived EV signaling may contribute to persistent inflammation and disease progression, whereas restoration of balanced EV-mediated communication may represent a promising therapeutic strategy (52). Collectively, these findings demonstrate that endogenous EVs function as central regulators of immune modulation, epithelial repair, and microbiota-host interactions. Their multifaceted biological activities provide a strong mechanistic foundation for the development of engineered EVs as therapeutic platforms for IBD and colitis-associated colorectal cancer (53).

### 2.4 Advantages of native EVs as therapeutic platforms

Growing understanding of EV biology has stimulated considerable interest in their application as therapeutic agents and drug delivery vehicles (32). Compared with conventional nanocarriers, such as liposomes and polymeric nanoparticles, native EVs possess several unique biological advantages that make them particularly attractive for the treatment of inflammatory diseases and cancer. First, native EVs can exhibit favorable biocompatibility and relatively low immunogenicity, particularly when they are derived from well-characterized, non-immunostimulatory donor cells. Most sources, including autologous or allogeneic MSC-derived native EVs, exhibit low immunogenicity in multiple experimental models. Their membranes express “self” markers, including cluster of differentiation 47 (CD47), which reduce recognition and clearance by the mononuclear phagocyte system, thereby prolonging circulation time and minimizing unwanted immune activation (54). These characteristics are especially advantageous for chronic diseases such as IBD that require repeated therapeutic administration (53). However, native EVs derived from tumor cells or certain xenogeneic sources may harbor immunoactive molecules and elicit immune responses. Moreover, EV surface proteins and their cargo, including exogenously introduced modifications or loaded molecules, can modulate the resultant immunological phenotype. Second, native EVs have been reported to cross certain biological barriers, including the blood–brain barrier and placental barrier, in selected experimental models, and may accumulate in inflamed tissues or solid tumors (55). However, EV biodistribution, tissue penetration, and cellular uptake are strongly influenced by EV source and subtype, surface composition, isolation method, dose, route of administration, and the pathological context. Their natural tissue tropism is therefore variable rather than universal, but can be further enhanced through engineering strategies, which may improve target-tissue delivery and reduce off-target exposure but require rigorous *in vivo* validation (56). Nonetheless, intrinsic ‘homing’ and barrier-penetrating capacities are not universal features of all native EVs and vary with EV origin, surface composition, isolation purity, and administration route (57). Third, the phospholipid bilayer of native EVs can facilitate biological-barrier penetration and provide an effective protective environment for encapsulated biomolecules. It should be noted that such penetration properties are variable, relying on the EV source, isolation protocol, administration route, and disease model investigated. Therapeutic proteins, mRNAs, miRNAs, and other nucleic acids can be protected, at least in part, from degradation by circulating nucleases, proteases, and other enzymatic factors, thereby potentially improving cargo stability, prolonging biological half-life, and enhancing delivery efficiency (31, 58, 59). Finally, unlike many synthetic nanocarriers, native EVs possess intrinsic biological activity. In addition to functioning as drug delivery vehicles, native EVs can directly regulate immune responses, inhibit inflammation, promote angiogenesis, suppress fibrosis, and facilitate tissue regeneration through their endogenous cargo (51). For example, mesenchymal stem cell-derived native EVs are naturally enriched with anti-inflammatory cytokines and regenerative miRNAs that contribute to immunomodulation and tissue repair (60). Nevertheless, their cargo composition and therapeutic activity can vary with MSC tissue source, donor characteristics, culture conditions, and EV isolation procedures (61, 62). In summary, the excellent biocompatibility, efficient penetration of biological barriers, cargo protection, natural targeting capability, and intrinsic therapeutic activity of native EVs distinguish them from conventional delivery systems and establish them as highly promising platforms for next-generation therapies. These unique advantages have driven increasing interest in engineering EVs to further enhance their targeting specificity, cargo-loading capacity, and therapeutic efficacy for IBD and CRC.

## 3 Engineering strategies for therapeutic EVs

It is necessary to differentiate the endogenous functions of native EVs from engineered EV-driven therapeutic mechanisms. Although native EVs possess intrinsic therapeutic properties, their native cargo composition, targeting specificity, and drug-loading capacity are often insufficient to achieve optimal therapeutic efficacy. Advances in bioengineering have enabled the development of engineered EVs with enhanced biological functions through modifications to their cargo, surface characteristics, and delivery systems (63). These engineering strategies can be broadly categorized into internal engineering, surface engineering, and hybrid EV formulations, which collectively improve targeting efficiency, cargo loading, stability, and therapeutic performance (**Figure 2**).

**Figure 2:**
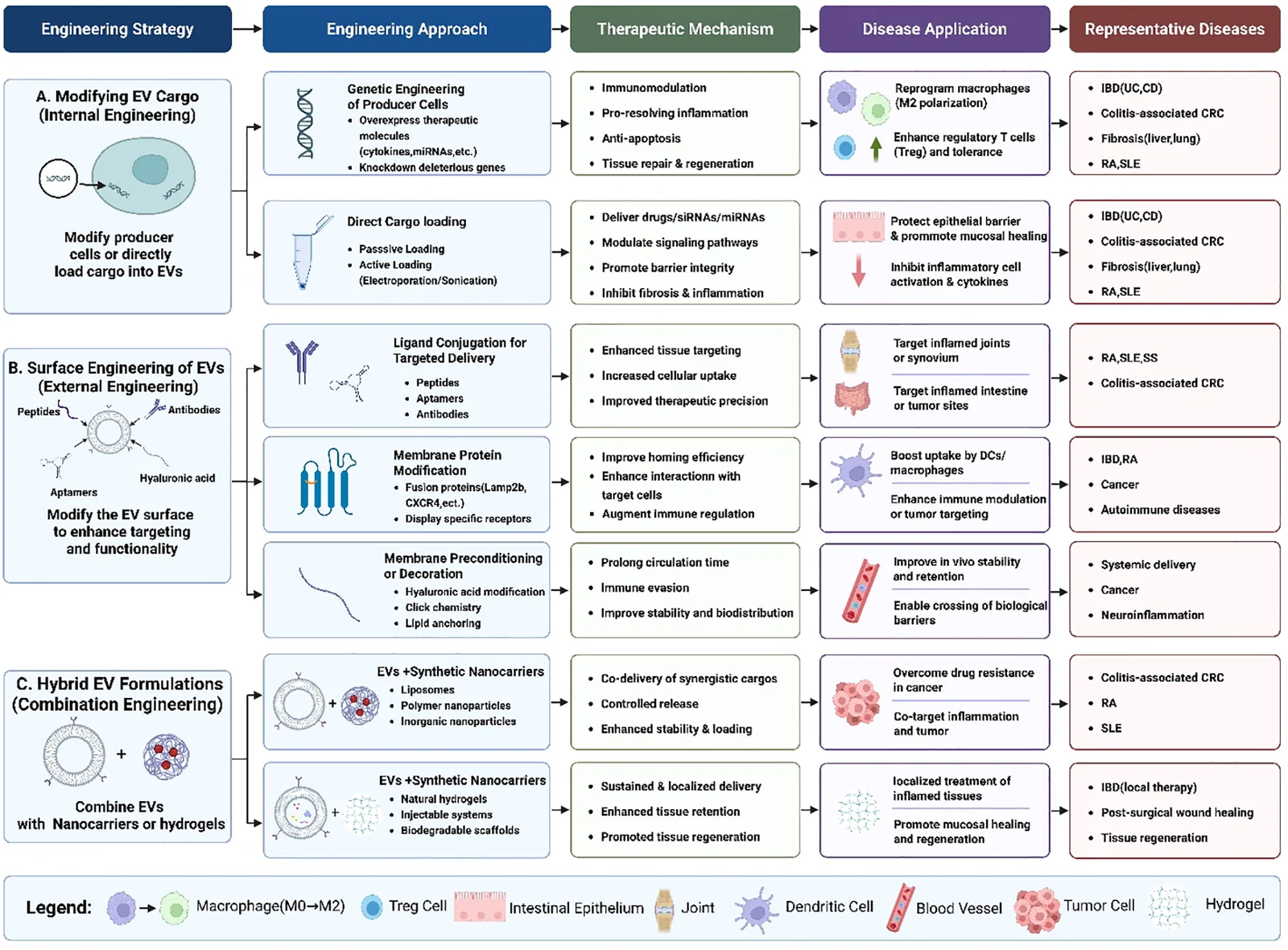
Engineering strategies of extracellular vesicles (EVs) and their therapeutic applications. Engineered EVs are generated through three major approaches: **(A)** internal cargo engineering, **(B)** surface engineering, and **(C)** hybrid EV formulations. Internal cargo engineering modifies EV cargo through genetic engineering of parental cells or direct loading of therapeutic agents. Surface engineering enhances targeting and biological activity via ligand conjugation or membrane protein modification. Hybrid EV formulations combine EVs with nanocarriers or hydrogels to improve cargo loading, stability, and localized delivery. These engineering strategies enhance immunomodulation, tissue repair, targeted delivery, and controlled therapeutic release, supporting their applications in inflammatory bowel disease (IBD), colitis-associated colorectal cancer (CAC), rheumatoid arthritis (RA), systemic lupus erythematosus (SLE), Sjögren’s syndrome (SS), fibrosis, neuroinflammatory disorders, and tissue regeneration.

### 3.1 Internal engineering: modifying EV cargo

Internal engineering aims to enhance the therapeutic efficacy of EVs by modifying their molecular cargo before or after EV biogenesis. This can be achieved through genetic engineering of parental cells or direct loading of purified EVs with therapeutic agents.

#### 3.1.1 Genetic engineering of parental cells

Genetic engineering of parental cells represents one of the most widely adopted strategies for producing therapeutic EVs. In this approach, donor cells, including MSCs and immune cells, are genetically modified to overexpress therapeutic proteins or nucleic acids, which are subsequently packaged into newly formed EVs during vesicle biogenesis (35). Compared with post-isolation cargo loading, genetic engineering enables the continuous production of engineered EVs while preserving their native membrane composition, biological stability, and intrinsic targeting properties (54). Consequently, this strategy has become an important platform for enhancing the therapeutic efficacy of EVs by enriching them with bioactive molecules that modulate inflammation, promote tissue regeneration, regulate gene expression, and improve targeted drug delivery. One of the most extensively investigated applications involves enriching EVs with anti-inflammatory cytokines. Genetic modification of parental cells to overexpress cytokines such as interleukin-10 (IL-10) and transforming growth factor-β (TGF-β) generates EVs that contain potent immunoregulatory molecules and can be delivered directly to inflamed tissues (64). For example, IL-10-overexpressing MSC-derived EVs have demonstrated superior therapeutic efficacy compared with native EVs in experimental models of IBD, arthritis, and graft-versus-host disease by promoting macrophage polarization toward the anti-inflammatory M2 phenotype while suppressing excessive T-cell activation and inflammatory cytokine production (65–68). These findings highlight the ability of genetically engineered EVs to reshape the immune microenvironment and restore immune homeostasis in chronic inflammatory diseases. Beyond immunomodulation, genetic engineering has also been widely used to enhance the regenerative capacity of EVs by incorporating growth factors. Overexpression of vascular endothelial growth factor (VEGF), epidermal growth factor (EGF), and fibroblast growth factor (FGF) in parental cells like MSCs results in EVs with enhanced regenerative potential, capable of stimulating angiogenesis, promoting cellular proliferation and migration, and accelerating tissue repair in models of myocardial infarction, skin injury, and peripheral nerve damage (69–73). These studies demonstrate that engineered EVs can serve not only as delivery vehicles but also as active mediators of tissue regeneration. Another rapidly expanding application is the delivery of regulatory nucleic acids, particularly microRNAs (miRNAs) and small interfering RNAs (siRNAs), which regulate gene expression through post-transcriptional mechanisms. Genetic modification of donor cells like MSCs and tumor cells enables efficient packaging of these regulatory RNAs into EVs for targeted delivery to recipient cells. Representative studies have shown that EV-mediated co-delivery of Kirsten rat sarcoma viral oncogene homolog(KRAS) antisense oligonucleotides and retinoic acid-inducible gene-I (RIG-I) agonists effectively suppresses pancreatic cancer metastasis while enhancing anti-tumor immune responses (74). Similarly, hypoxia-preconditioned MSC-derived EVs enriched with miR-21 promote functional recovery following spinal cord injury through modulation of the JAK2/STAT3 signaling pathway, whereas endothelial cell-derived EVs carrying circWhsc1 stimulate cardiomyocyte proliferation and cardiac regeneration after myocardial infarction (75, 76). These findings illustrate the versatility of engineered EVs as gene delivery platforms capable of regulating diverse pathological processes. In addition to nucleic acids, therapeutic proteins can also be efficiently incorporated into EVs through genetic engineering of parental cells, such as MSCs and tumor cells. This strategy improves protein stability, bioavailability, and targeted delivery while overcoming several limitations associated with conventional protein therapeutics, including rapid degradation and poor tissue penetration (77). Notably, Choi and colleagues developed a blue-light-controlled protein-loading platform that selectively directs therapeutic proteins into either the EV lumen or its membrane, providing a highly versatile system for protein delivery (78). Furthermore, EV-based protein vaccines have emerged as promising candidates for cancer immunotherapy and the prevention of infectious diseases, highlighting the expanding applications of engineered EVs in precision medicine (79). In summary, genetic engineering of parental cells enables the stable incorporation of diverse therapeutic cargos, including cytokines, growth factors, regulatory RNAs, and proteins, into EVs while maintaining their natural membrane architecture and biological compatibility. Owing to its ability to generate EVs with enhanced immunomodulatory, regenerative, and gene-regulatory functions, this strategy has become one of the most powerful and versatile approaches for the development of next-generation EV-based therapeutics.

#### 3.1.2 Direct loading of therapeutic agents

Direct loading is an alternative engineering strategy in which therapeutic molecules are incorporated into purified EVs after isolation, thereby eliminating the need for genetic modification of parental cells and providing greater flexibility in cargo selection. Depending on the loading mechanism, direct loading approaches are broadly classified as passive or active. Passive loading is typically achieved through simple co-incubation of therapeutic compounds with donor cells or isolated EVs, allowing molecules to diffuse across the lipid bilayer according to concentration gradients (63, 80). This strategy is particularly suitable for hydrophobic compounds, such as curcumin, paclitaxel, and doxorubicin, which readily partition into the lipid membrane of EVs (81–83). Although passive loading is technically simple and largely preserves EV structural integrity, its loading efficiency is generally low and is highly dependent on the physicochemical properties of the therapeutic cargo (84). In contrast, active loading strategies transiently increase membrane permeability to facilitate more efficient encapsulation of therapeutic agents. Among these, electroporation is the most widely used technique for purified EVs, employing short electrical pulses to temporarily permeabilize the EV membrane and enable efficient loading of nucleic acids and small-molecule drugs, including siRNAs, miRNAs, curcumin, and paclitaxel (85). However, electroporation may induce aggregation of EVs or nucleic acids, requiring careful optimization of loading conditions to maintain vesicle integrity and therapeutic efficacy (86). Sonication represents another commonly used approach in which ultrasonic energy transiently disrupts the membrane of purified EVs, allowing therapeutic molecules to enter the vesicle lumen. Compared with passive loading and electroporation, sonication generally achieves higher loading efficiency, although excessive ultrasonic treatment may compromise membrane integrity and alter EV biological properties (82, 87). Similarly, extrusion enhances cargo encapsulation by mechanically forcing purified EVs and therapeutic agents through membrane filters, resulting in relatively uniform vesicle populations with high loading efficiency; however, excessive mechanical stress may alter membrane composition and biological activity (80). Another widely employed active loading strategy is saponin-assisted permeabilization for purified EVs, which temporarily increases membrane permeability through reversible pore formation by interacting with membrane cholesterol, thereby improving the encapsulation of hydrophilic molecules while largely preserving EV structural integrity (80, 88). Overall, direct loading strategies offer versatile, efficient methods for incorporating a wide range of therapeutic agents into EVs. The selection of an appropriate loading method depends on the physicochemical characteristics of the therapeutic cargo, the desired loading efficiency, and the need to preserve EV membrane integrity and biological function, making these approaches valuable tools for the development of engineered EV-based therapies.

### 3.2 Surface engineering of EVs

Surface engineering enhances the targeting specificity and therapeutic efficacy of EVs by modifying membrane-associated molecules without altering their endogenous cargo. Current strategies mainly include ligand conjugation and membrane protein engineering.

#### 3.2.1 Ligand conjugation for targeted delivery

Ligand conjugation is one of the most widely employed surface engineering strategies for enhancing the targeting specificity of EVs. By decorating the EV membrane with ligands that selectively recognize receptors overexpressed on diseased tissues, engineered EVs can achieve improved cellular uptake, enhanced tissue accumulation, and reduced off-target effects, thereby increasing therapeutic efficacy (89). A variety of targeting ligands, including peptides, antibodies, aptamers, and polysaccharides, have been developed to improve the precision of EV-based drug delivery. Among these, peptide ligands are the most extensively investigated because of their small size, high specificity, and ease of genetic engineering. Targeting peptides can be fused to the membrane proteins of MSC-EVs, such as lysosome-associated membrane protein-2B (LAMP-2B), thereby enabling their stable display on the EV surface and facilitating selective recognition of target tissues (35). One of the best-characterized examples is the cyclic arginine-glycine-aspartate (cRGD) peptide, which exhibits high affinity for αvβ3 integrin, a receptor that is highly expressed on activated endothelial cells in inflamed tissues and tumor vasculature. Consequently, cRGD-functionalized EVs have emerged as promising targeted delivery vehicles for inflammatory bowel disease (IBD) and colitis-associated colorectal cancer (CAC), in which αvβ3 integrin expression is markedly increased (90–92). Antibody-based targeting represents another important strategy for improving EV specificity. Surface modification with monoclonal antibodies or antibody-binding domains enables engineered EVs to selectively recognize inflammatory or tumor-associated antigens, thereby enhancing targeted drug delivery while minimizing systemic toxicity (93). For example, EVs engineered to display antibodies against human epidermal growth factor receptor 2 (HER2) or programmed death-ligand 1 (PD-L1) have demonstrated enhanced tumor localization and significantly improved anti-tumor efficacy in preclinical cancer models, highlighting their potential for precision cancer therapy (94, 95). In addition to peptides and antibodies, aptamers have attracted increasing attention as alternative targeting ligands. Aptamers are short single-stranded DNA or RNA molecules capable of binding target molecules with high affinity and specificity. Compared with antibodies, they offer several practical advantages, including lower immunogenicity, reduced production costs, improved chemical stability, and easier synthesis and modification, making them attractive candidates for both targeted diagnosis and therapeutic delivery via engineered EVs (96–98). Hyaluronic acid (HA) has also been widely investigated as a natural targeting ligand for MSC-EVs because of its strong affinity for cluster of differentiation 44(CD44), a receptor that is frequently overexpressed on inflammatory cells, intestinal epithelial cells, and numerous cancer cell types (99–101). Through HA–CD44 interactions, HA-functionalized EVs exhibit enhanced uptake by macrophages and other immune cells, thereby improving targeted delivery to inflamed tissues. Notably, HA-modified mesenchymal stem cell-derived EVs have demonstrated enhanced anti-inflammatory activity and improved macrophage targeting compared with unmodified EVs, supporting their therapeutic potential for inflammatory diseases such as IBD (100, 102). Collectively, ligand conjugation significantly enhances the targeting capability of engineered EVs by exploiting disease-specific receptor–ligand interactions. Continued development of peptide-, antibody-, aptamer-, and polysaccharide-based targeting strategies is expected to improve delivery precision, increase therapeutic efficacy, and facilitate the clinical translation of EV-based therapies for inflammatory disorders and cancer.

#### 3.2.2 Membrane protein modification

Membrane protein modification is another important surface engineering strategy that enhances the targeting specificity, cellular uptake, and therapeutic efficacy of EVs. Unlike ligand conjugation, which attaches exogenous targeting molecules to the EV surface, membrane protein engineering leverages endogenous EV membrane proteins as scaffolds to display therapeutic peptides, proteins, or receptors, thereby preserving membrane integrity while enhancing selective interactions with target cells (103). This strategy enables engineered EVs to achieve greater targeting precision and biological functionality without compromising their intrinsic physicochemical properties. One of the most widely adopted approaches for MSC-EVs involves the genetic fusion of targeting peptides or therapeutic proteins with naturally abundant EV membrane proteins, including lysosome-associated membrane protein-2B (Lamp2b), tetraspanins (CD63, CD81, and CD9), platelet-derived growth factor receptor (PDGFR), prostaglandin F2 receptor negative regulator (PTGFRN), syntenin-1, and C-X-C chemokine receptor type 4 (CXCR4) (103, 104). These membrane proteins serve as anchoring scaffolds that facilitate stable surface display of targeting moieties, thereby improving EV recognition, cellular internalization, and selective delivery to diseased tissues. Beyond fusion protein engineering, engineered EVs can also be modified to display functional membrane receptors that act as biological decoys, neutralizing pathogenic signaling molecules. This approach has shown considerable promise for treating inflammatory diseases by sequestering pro-inflammatory cytokines before they interact with their cellular receptors. For example, engineered EVs co-expressing tumor necrosis factor receptor 1 (TNFR1) and interleukin-6 signal transducer (IL-6ST) effectively inhibit tumor necrosis factor-alpha (TNF-α) and interleukin-6(IL-6) signaling pathways and have demonstrated superior therapeutic efficacy compared with clinically approved biologics in experimental models of systemic, neuroinflammatory, and intestinal inflammation (105). These findings highlight the potential of receptor-displaying EVs as next-generation biologics capable of simultaneously targeting multiple inflammatory pathways. Overall, membrane protein modification complements ligand conjugation by providing a versatile platform for engineering multifunctional EVs with enhanced targeting capabilities and therapeutic activity. As advances in membrane protein engineering continue, this strategy is expected to facilitate the development of highly selective EV-based therapeutics for inflammatory diseases, including IBD, as well as cancer and other immune-mediated disorders.

### 3.3 Hybrid EV formulations

Hybrid EV formulations represent an emerging engineering strategy that integrates the intrinsic biological properties of EVs with synthetic biomaterials to overcome the limitations of either platform alone. Although native EVs possess excellent biocompatibility, low immunogenicity, and inherent targeting capabilities, their clinical translation remains constrained by limited production yield, cargo-loading efficiency, and *in vivo* stability. Conversely, synthetic delivery systems offer high loading capacity and tunable physicochemical properties but often suffer from poor biocompatibility, rapid clearance, and inadequate tissue specificity. By combining these complementary features, hybrid EV systems aim to enhance delivery efficiency, therapeutic stability, targeting precision, and overall treatment efficacy (106–108). One of the most extensively investigated approaches involves integrating EVs with synthetic nanoparticles. Nanoparticles possess favorable characteristics, including high drug-loading capacity, controlled drug release, and versatile surface modification; however, their clinical application is frequently limited by rapid immune clearance and insufficient accumulation at target tissues (106, 107). Coating synthetic nanoparticles with membranes derived from EV–nanoparticle hybrids combines the immune-evasive and targeting properties of EVs with the engineering flexibility of nanomaterials, thereby improving circulation time, enhancing tissue-specific delivery, reducing systemic toxicity, and increasing therapeutic efficacy (108–110). These hybrid nanoplatforms have demonstrated considerable promise for targeted drug delivery and precision therapy in both inflammatory diseases and cancer. Another promising strategy involves constructing EV-loaded biomaterial-based delivery systems, particularly hydrogels, to achieve localized and sustained therapeutic release. Hydrogels possess excellent biocompatibility, high water content, and tunable mechanical properties, enabling them to serve as protective reservoirs that preserve EV bioactivity while preventing rapid degradation after administration (111). Furthermore, the porous three-dimensional architecture of hydrogels enables controlled and prolonged EV release at the target site, thereby extending therapeutic retention and improving treatment outcomes. Consequently, EV-loaded hydrogels have shown significant potential for localized treatment of inflammatory disorders, tissue regeneration, wound healing, and regenerative medicine applications (112, 113). Collectively, advances in internal cargo engineering, surface functionalization, and hybrid EV formulations have substantially expanded the therapeutic potential of EVs by improving cargo loading efficiency, targeting specificity, biological stability, and controlled drug release. These complementary engineering strategies have enabled the development of multifunctional EV-based delivery platforms with enhanced therapeutic efficacy for inflammatory diseases, including IBD, CRC, and other immune-mediated disorders. The major EV engineering strategies, representative therapeutic cargos, mechanisms of action, and their corresponding therapeutic advantages are summarized in **Table 1**.

**Table 1:** Major engineering strategies used to enhance extracellular vesicle (EV) therapeutic efficacy and their translational considerations.

Engineering strategy	EV source	Engineering method	Representative cargo/modification	Representative disease application	Therapeutic advantages	Main limitation	Level of evidence	Refs.
Internal engineering (genetic modification)	MSCs, immune cells	Genetic modification of parental cells	IL-10, TGF-β	IBD, autoimmune diseases	Potent immunomodulation and prolonged therapeutic activity	Variable transgene expression; manufacturing complexity	Preclinical	(65, 68)
	MSCs	Growth factor overexpression	VEGF, EGF, FGF	Tissue repair and regeneration	Promotes angiogenesis and tissue repair	Risk of excessive angiogenesis	Preclinical	(72, 73)
	MSCs, tumor cells	miRNA/siRNA overexpression	miR-21, therapeutic siRNAs	IBD, CRC	Precise gene regulation	Off-target effects and loading variability	Preclinical	(74–76)
	MSCs, tumor cells	Therapeutic protein overexpression	Enzymes, antibodies	Cancer, inflammatory diseases	Improved protein stability and targeted delivery	Low protein loading efficiency	Preclinical	(77–79)
Internal engineering (direct cargo loading)	Purified EVs	Passive incubation	Curcumin, paclitaxel, doxorubicin	Inflammation, cancer	Simple preparation preserves EV integrity	Low loading efficiency	Preclinical	(63, 80–84)
	Purified EVs	Electroporation	siRNA, miRNA, curcumin	Inflammation, cancer	Efficient nucleic acid loading	Cargo aggregation and membrane damage	Preclinical	(85, 86)
	Purified EVs	Sonication	Small-molecule drugs	Cancer	High loading efficiency	May disrupt membrane integrity	Preclinical	(82, 87)
	Purified EVs	Extrusion	Chemotherapeutic drugs	Cancer	Uniform drug encapsulation	Possible alteration of EV membrane proteins	Preclinical	(80)
	Purified EVs	Saponin-assisted permeabilization	Catalase, porphyrin	Oxidative stress, inflammation	Efficient loading of hydrophilic molecules	Residual saponin toxicity	Preclinical	(80, 88)
Surface engineering	MSC-EVs	Peptide conjugation	cRGD	IBD, CRC	Enhanced targeting to inflamed or tumor tissues	Limited peptide stability	Preclinical	(35, 91)
	Various EVs	Antibody conjugation	HER2, PD-L1 antibodies	CRC	Improved targeting specificity	High production cost	Preclinical	(94, 95)
	Various EVs	Aptamer conjugation	DNA/RNA aptamers	Cancer	Highly specific targeting	Susceptible to nuclease degradation	Preclinical	(96–98)
	MSC-EVs	Hyaluronic acid modification	HA	IBD	Improved macrophage targeting through CD44	Limited tissue penetration	Preclinical	(100, 102)
	MSC-EVs	Membrane protein engineering	Lamp2b, CXCR4, PDGFR	IBD, CRC	Enhanced tissue-specific delivery	Complex vector construction	Preclinical	(103, 104)
	Engineered EVs	Receptor display	TNFR1, IL-6ST	Inflammatory diseases	Neutralizes inflammatory cytokines	Limited *in vivo* validation	Preclinical	(105)
Hybrid EV formulations	EV–nanoparticle hybrids	Membrane coating	Nanoparticles	IBD, CRC	Increased drug loading and prolonged circulation	Complex manufacturing	Preclinical	(106–110)
	EV-loaded biomaterials	Hydrogel encapsulation	Hydrogels	IBD, wound healing	Sustained local release and prolonged retention	Limited large-animal validation	Preclinical	(111–113)

EVs, extracellular vesicles; IL-10, interleukin-10; TGF-β, transforming growth factor-beta; VEGF, vascular endothelial growth factor; EGF, epidermal growth factor; FGF, fibroblast growth factor; miRNA, microRNA; siRNA, small interfering RNA; cRGD, cyclic Arg-Gly-Asp peptide; HER2, human epidermal growth factor receptor 2; PD-L1, programmed death-ligand 1; CD44, cluster of differentiation 44; HA, hyaluronic acid; LAMP2B, lysosome-associated membrane protein 2B; CXCR4, C-X-C chemokine receptor type 4; PDGFR, platelet-derived growth factor receptor; PTGFRN, prostaglandin F2 receptor negative regulator; TNFR1, tumor necrosis factor receptor 1; IL-6ST, interleukin-6 signal transducer (also known as gp130); NPs, nanoparticles.

### 3.4 Matching EV engineering strategies to the therapeutic requirements of IBD and CAC

The selection of an EV engineering strategy should be guided primarily by the biological barrier and therapeutic objective to be addressed rather than by engineering feasibility alone. In IBD, immune dysregulation, epithelial barrier disruption, and gut microbiota imbalance are closely interconnected components of disease pathogenesis, suggesting that EV platforms may need to address one or more of these processes simultaneously (114–116). Accordingly, engineering strategies can be tailored to the predominant pathological feature, such as incorporating immunomodulatory cargos to suppress excessive inflammatory activation, targeting epithelial repair pathways to restore barrier integrity, or improving gastrointestinal stability and cargo delivery for locally acting or microbiota-directed therapies. Several studies support this disease-oriented design principle. Kang et al. demonstrated that hyaluronic acid-functionalized plant-derived EVs could simultaneously modulate inflammatory macrophage responses and promote intestinal epithelial regeneration, resulting in improved therapeutic effects in experimental colitis (114). Similarly, EV-based approaches targeting epithelial repair have demonstrated the capacity to enhance intestinal barrier integrity, supporting the use of EV engineering strategies tailored to epithelial dysfunction (116). Targeted delivery can further improve therapeutic specificity; for example, IL-10-loaded EVs directed toward inflammatory macrophages have been investigated for reducing oxidative stress and pro-inflammatory responses in experimental colitis. Collectively, these findings suggest that EV engineering should be matched to the dominant pathological mechanism and desired therapeutic outcome rather than optimized solely for cargo-loading efficiency or technical feasibility. However, the available evidence is substantially stronger for IBD than for CAC. Most studies evaluating disease-oriented EV engineering have focused on experimental colitis or general intestinal inflammation. In contrast, direct evidence demonstrating that a particular engineering strategy is optimal for inflammation-associated CRC remains limited. This distinction is important because CAC develops within a persistent inflammatory, immune, stromal, and microbiota-altered environment that may influence EV biodistribution, cellular uptake, therapeutic efficacy, and safety (117). Therefore, engineering strategies that are effective for controlling inflammation or restoring epithelial integrity in IBD cannot automatically be assumed to provide equivalent benefits in CAC. Future studies should compare different EV engineering strategies in clinically relevant inflammation-associated tumor models and evaluate their effects on immune regulation, epithelial and tumor-cell targeting, microbiota interactions, biodistribution, and safety. Such comparative studies will help establish rational, disease-specific design principles for engineered EV therapies across the IBD-to-CAC continuum.

## 4 Therapeutic applications of engineered EVs in IBD

IBD is characterized by excessive immune activation, disruption of intestinal epithelial integrity, and dysbiosis of the gut microbiota. Although conventional therapies have improved disease management, limitations, including incomplete response, systemic adverse effects, and loss of therapeutic efficacy, remain major challenges. EVs provide a versatile therapeutic platform owing to their intrinsic biocompatibility, efficient intercellular communication capacity, and ability to deliver diverse biological cargos, including nucleic acids, proteins, and small molecules. By incorporating specific therapeutic cargos or modifying EV surfaces, engineered EVs can regulate inflammatory signaling, restore epithelial barrier function, and remodel intestinal microbial ecosystems. Therefore, current therapeutic applications of engineered EVs in IBD mainly focus on three complementary strategies: immunomodulation, intestinal barrier regeneration, and microbiota regulation.

### 4.1 Immunomodulation and anti-inflammatory effects

#### 4.1.1 Delivery of immunoregulatory cargos to immune cells

Excessive activation of immune cells, particularly macrophages and T lymphocytes, is a central driver of chronic intestinal inflammation in IBD. Engineered EVs can serve as potential efficient carriers for delivering anti-inflammatory microRNAs, proteins, and other immunoregulatory molecules to immune cells, thereby suppressing inflammatory signaling and restoring immune homeostasis. Among the therapeutic cargos investigated, microRNAs represent one of the most promising approaches because of their ability to regulate multiple inflammatory pathways simultaneously. However, this pleiotropic activity also creates a risk of context-dependent and off-target effects; therefore, miRNA selection should be guided by the dominant inflammatory endotype, target-cell population, and the intended therapeutic objective. Studies have demonstrated that EV-mediated delivery of miR-146a effectively suppresses macrophage activation by inhibiting the NF-κB signaling pathway, thereby reducing the production of pro-inflammatory cytokines, including TNF-α and IL-6 (118). In addition, engineered EVs carrying miR-146a can inhibit Th1 cell activation by modulating the AKT/NF-κB pathway, thereby reducing excessive intestinal immune responses and limiting tissue damage. miR-146a is prioritized in this context not because it has been conclusively shown to be more efficacious than all alternative miRNAs in IBD, as direct head-to-head comparisons remain unavailable, but because it acts as a well-characterized negative-feedback regulator of Toll-like receptor (TLR)/IL-1 receptor signaling. By targeting key proximal mediators, particularly Interleukin-1 receptor-associated kinase 1(IRAK1) and TNF receptor-associated factor 6(TRAF6), miR-146a can attenuate NF-κB-driven inflammatory amplification in myeloid cells (119). This mechanism provides a biologically coherent rationale for its use in IBD, in which excessive innate immune activation and NF-κB signaling are central pathogenic features. In contrast, miR-155 is broadly induced in activated macrophages and T cells and regulates a wider network of immune-associated targets, including Suppressor of Cytokine Signaling 1(SOCS1), SH2-domain-containing inositol-5-phosphatase 1(SHIP1), and B-cell lymphoma 6(BCL6) (120). Although this broader activity may be advantageous when extensive immune reprogramming is required, miR-155 is often associated with enhanced macrophage activation and Th1/Th17-polarized responses. Its functional consequences are therefore strongly dependent on the target cell, inflammatory context, and whether a mimic or inhibitor is delivered (121, 122). For example, miR-155 overexpression in an inflammatory setting may increase RAR-related orphan receptor gamma t(RORγt), IL-17, and IL-6 expression, whereas miR-146a-enriched MSC-EVs have been associated with increased Forkhead box P3 (FoxP3), IL-10, and TGF-β expression (122). Accordingly, miR-146a may be a more rational first-choice cargo for EV-mediated suppression of NF-κB-dominant intestinal inflammation, while miR-155 modulation may be better considered for biomarker-defined settings and requires more stringent assessment of cell-specific effects (123). Importantly, miR-146a itself is not free from potential non-target effects, because it may regulate additional transcripts involved in immune-cell survival, antigen presentation, and tissue repair. Therefore, the therapeutic index of miR-146a-EVs should be established through target-cell uptake studies, transcriptomic or proteomic off-target assessment, dose-response analyses, and evaluation of host-defense competence in relevant IBD models. Beyond microRNA delivery, engineered EVs can also transport functional proteins or cytokine regulators to immune cells, enabling potentially more precise, but still product- and target-cell-dependent, immune modulation (124). These approaches highlight the potential of engineered EVs to regulate inflammatory pathways while potentially reducing selectively, rather than eliminating, avoiding the systemic toxicity associated with conventional immunosuppressive therapies.

#### 4.1.2 Promotion of macrophage M2 polarization and inflammatory resolution

Macrophage polarization plays a critical role in determining the outcome of intestinal inflammation. During the early inflammatory phase, M1 macrophages promote pathogen clearance through the secretion of inflammatory mediators, whereas M2 macrophages contribute to the resolution of inflammation, tissue repair, extracellular matrix remodeling, and angiogenesis during the recovery phase (125). Therefore, promoting the transition of macrophages from a pro-inflammatory M1 phenotype to an anti-inflammatory M2 phenotype represents an attractive therapeutic strategy for IBD. EVs have been shown to regulate macrophage polarization by altering surface marker expression and cytokine production. Treatment with EVs can reduce M1-associated inflammatory markers, decrease secretion of IL-6 and IL-1β, increase expression of the M2 marker CD206, and promote macrophage polarization toward an anti-inflammatory phenotype, particularly M2b differentiation (114). Furthermore, under inflammatory conditions induced by lipopolysaccharide (LPS) and interferon-γ, EV treatment suppresses the expression of the M1-associated marker human leukocyte antigen-DR alpha chain(HLA-DRα), suggesting that EVs can counteract inflammatory macrophage activation (126). Recent advances in plant- and microbial-derived EV engineering have further expanded this therapeutic strategy. Oral administration of polydeoxyribonucleotide (PDRN)-loaded tea-derived EVs activates the cAMP/HIF-1α signaling pathway, promoting M2 macrophage polarization. In DSS-induced colitis models, PDRN-EVs significantly reduced inflammatory responses and improved intestinal barrier function, demonstrating the therapeutic potential of orally administered engineered EVs for intestinal inflammatory diseases (127).

### 4.2 Restoration of intestinal barrier integrity

The intestinal epithelial barrier represents the first line of defense against luminal antigens, microorganisms, and inflammatory stimuli. Disruption of epithelial integrity is a hallmark of IBD and contributes to persistent immune activation through increased microbial translocation and mucosal immune stimulation (128). Engineered EVs can promote epithelial repair by enhancing intestinal epithelial cell (IEC) proliferation, stimulating wound healing pathways, and restoring tight junction integrity (129).

#### 4.2.1 Promotion of intestinal epithelial cell proliferation and wound repair

Loss of IEC regenerative capacity is closely associated with impaired mucosal healing in IBD. Engineered EVs can enhance epithelial regeneration by delivering functional microRNAs, proteins, and signaling molecules that activate repair-associated pathways. Dendritic cell-derived extracellular vesicles containing miR-146b have been shown to improve intestinal barrier function by regulating the ubiquitination-related protein Siah2 and modulating tumor necrosis factor receptor-associated factor (TRAF)-associated inflammatory signaling in DSS-induced colitis models (130). In addition, extracellular vesicles derived from Lactobacillus rhamnosus GG promote IEC proliferation, re-epithelialization, and angiogenesis by delivering miR-21-5p, thereby accelerating intestinal wound healing (131). Immune-cell-derived EVs also participate in endogenous repair processes. EV-associated Annexin A1 activates epithelial wound repair pathways, and increased levels of Annexin A1-containing EVs have been detected in patients with active IBD, suggesting their involvement in compensatory restoration of barrier integrity (132). Moreover, several studies have demonstrated that EVs can activate Wnt/β-catenin signaling, enhancing intestinal stem cell activity and epithelial regeneration capacity (133, 134).

#### 4.2.2 Restoration of tight junction integrity

Tight junction (TJ) complexes, including zonula occludens-1 (ZO-1), occludin, and claudins, regulate epithelial permeability and maintain intestinal barrier function. TJ disruption facilitates penetration of microbial products and inflammatory mediators into the mucosa, thereby exacerbating intestinal inflammation. To improve targeted intestinal delivery, researchers developed double-coated extracellular vesicles (DCEVs) with enhanced gastrointestinal stability. For example, in human colon adenocarcinoma cell line-2 (Caco-2) monoculture and Caco-2/human acute monocytic leukemia cell line (THP-1) coculture models, DCEVs significantly increased expression of ZO-1, occludin, and claudin-1, indicating improved epithelial barrier integrity (130). Similarly, MSC-derived EVs engineered with mitochondria-targeted bismuth nanozyme (Ce-TPP) and probiotic inulin improved intestinal barrier function in DSS-induced colitis by restoring tight junction expression and correcting microbiota imbalance (135). Platelet-derived EVs have also been shown to release cellular prion protein, maintaining tight junction organization and protecting epithelial junction complexes during intestinal inflammation (136).

#### 4.2.3 Modulation of gut microbiota through engineered EVs

Gut microbiota dysbiosis contributes significantly to IBD pathogenesis by promoting excessive immune activation, metabolic imbalance, and epithelial dysfunction. Engineered EVs provide a unique opportunity to regulate microbial communities by delivering functional molecules directly to intestinal microorganisms or modifying host–microbe interactions. To enhance gastrointestinal stability and biological delivery, researchers fused a cell-penetrating peptide (TAT) with green fluorescent protein (GFP) for EV loading. This strategy improved cargo incorporation, promoted cellular uptake, and protected biological molecules from gastrointestinal degradation. Furthermore, oral administration of EV-delivered anti-TNF-α nanobodies (VHHm3F) significantly reduced inflammatory responses in models of acute ulcerative colitis compared with free nanobody administration, demonstrating the potential of engineered EVs for oral biologic delivery (137). Beyond therapeutic cargo delivery, endogenous intestinal EVs can directly regulate bacterial behavior. Intestinal EV-derived microRNAs, including miR-515-5p and miR-1226-5p, can enter pathogenic bacteria such as Fusobacterium nucleatum and Escherichia coli, regulate bacterial gene expression, and influence microbial growth (138). In addition, probiotic-derived EV-like membrane vesicles represent a promising microbiota-targeting strategy. Vesicles derived from *Lactobacillus helveticus R0052* restored DSS-induced microbial dysbiosis, increased bacterial diversity, and corrected associated metabolic disturbances, highlighting their potential as microbiome-modulating therapeutics for IBD (139). The representative engineered EV-based therapeutic strategies for IBD, including immune modulation, epithelial barrier restoration, and microbiota regulation, are summarized in **Table 2**. Collectively, engineered EVs alleviate intestinal inflammation by modulating immune responses, restoring epithelial barrier integrity, and maintaining gut microbial homeostasis. However, persistent inflammation remains a major driver of CRC development. Chronic inflammatory signaling promotes genetic instability, oxidative stress, epithelial dysplasia, angiogenesis, and remodeling of the tumor microenvironment, ultimately facilitating malignant transformation. The major molecular pathways linking chronic intestinal inflammation to CRC are illustrated in **Figure 3**, providing the mechanistic basis for the therapeutic application of engineered EVs in IBD-associated CRC.

**Table 2:** Representative engineered extracellular vesicle-based therapeutic strategies for IBD, including therapeutic rationale and potential limitations.

EV source/engineering strategy	Therapeutic cargo or modification	Target cell/pathway	Mechanism of action	Therapeutic rationale	Potential limitation	Therapeutic outcome	Refs.
Engineered EVs (miR-146a-loaded)	miR-146a	Macrophages; NF-κB signaling	Targets TRAF6 and IRAK1 and suppresses NF-κB activation	Suitable for restraining excessive inflammatory signaling and reducing pro-inflammatory cytokine production	Broad regulatory effects and target-cell specificity require further validation; direct comparative efficacy with other anti-inflammatory miRNAs remains unclear	Reduced TNF-α, IL-6, and IL-1β production and attenuation of intestinal inflammation	(123)
EV-mediated immunomodulation	EV-associated immunoregulatory molecules	Macrophages	Regulation of macrophage polarization	Potentially useful for shifting macrophages toward a resolution-associated phenotype and reducing excessive inflammatory activity	Effects may depend on macrophage activation state and disease context; durability and specificity require further investigation	Increased CD206 expression and reduced IL-6/IL-1β secretion	(114)
EVs under inflammatory stimulation	EV-mediated immune modulation	M1 macrophages	Suppression of HLA-DRα expression	May help reduce excessive inflammatory macrophage activation	Limited information regarding target specificity, durability, and comparative efficacy	Reduced inflammatory macrophage activation	(126)
Tea-derived EVs	Polydeoxyribonucleotide (PDRN) loading	Macrophages; cAMP/HIF-1α pathway	Activation of macrophage polarization pathways	Potentially promotes inflammation resolution through macrophage reprogramming and may complement barrier-repair effects	Source- and dose-dependent activity; translational evidence and long-term safety remain limited	Enhanced M2 polarization and improved DSS-induced colitis	(127)
Dendritic cell-derived exosomes	miR-146b	IEC inflammatory signaling	Regulation of Siah2/TRAF-associated signaling and epithelial repair	May be advantageous when suppression of inflammation and restoration of epithelial function are both therapeutic objectives	Limited comparative efficacy data and insufficient evidence regarding target-cell specificity and off-target effects	Improved intestinal barrier function and reduced colitis severity	(130)
*Lactobacillus rhamnosus* GG-derived EVs	miR-21-5p	Intestinal epithelial cells	Promotion of IEC proliferation and angiogenesis	Potentially useful for epithelial repair and wound healing	miR-21-5p has context-dependent effects on inflammation and proliferation; broader effects require further evaluation	Accelerated epithelial wound healing	(131)
Immune-cell-derived EVs	Annexin A1	Epithelial repair pathways	Activation of wound-repair circuits	Suitable for promoting endogenous epithelial repair and restoration of barrier integrity	Mechanistic specificity and comparative efficacy relative to other repair-associated cargos remain insufficiently defined	Restoration of intestinal barrier integrity	(132)
Engineered EVs	Regulatory cargos	Wnt/β-catenin signaling	Activation of epithelial regenerative pathways	Potentially useful when impaired epithelial regeneration is a major therapeutic target	Wnt/β-catenin signaling has context-dependent effects, and excessive activation may require careful control	Enhanced intestinal epithelial regeneration	(133, 134)
MSC-derived EVs	Ce-TPP nanozyme and inulin	Tight-junction proteins and gut microbiota	Restoration of barrier function and microbiome balance	Addresses multiple interconnected components of IBD, including epithelial dysfunction, oxidative stress, and microbiota imbalance	Increased engineering complexity may affect manufacturing consistency, stability, and translational scalability	Increased ZO-1/Occludin/Claudin-1 expression and reduced inflammation	(135)
Platelet-derived exosomes	Cellular prion protein	Tight-junction complexes	Stabilization of epithelial junctions	Potentially suitable for barrier-restoration strategies in which epithelial permeability is a major therapeutic target	Limited information on target specificity, dose optimization, and long-term efficacy	Protection of intestinal barrier integrity	(136)
Engineered oral EV delivery platform	TAT-GFP fusion system and anti-TNF-α nanobody	Intestinal inflammatory environment	Enhanced cargo protection and oral delivery efficiency	Particularly relevant to IBD because improved gastrointestinal stability and local intestinal exposure may enhance therapeutic efficacy while limiting systemic exposure	Oral delivery efficiency, gastrointestinal stability, biodistribution, and manufacturing reproducibility require further validation	Reduced experimental colitis severity	(137)
Intestinal EV-derived miRNAs	miR-515-5p and miR-1226-5p	Gut bacteria (*F. nucleatum*, *E. coli*)	Regulation of bacterial gene expression	Provides a potential microbiota-directed strategy for modulating host–microbiota interactions	Bacterial specificity and effects on broader microbial communities require further investigation	Modulation of bacterial growth and host–microbiota interaction	(138)
*Lactobacillus helveticus* R0052 membrane vesicles	Microbial EV-like vesicles	Gut microbiota	Microbiome remodeling	Potentially useful for restoring microbial diversity and metabolic homeostasis	Microbiome responses may vary between individuals; mechanisms, dose optimization, and translational consistency remain to be established	Increased microbial diversity and correction of metabolic imbalance	(139)

EV, extracellular vesicle; EVs, extracellular vesicles; IBD, inflammatory bowel disease; miR, microRNA; NF-κB, nuclear factor kappa B; TNF-α, tumor necrosis factor-alpha; IL-1β, interleukin-1 beta; IL-6, interleukin-6; HLA-DRα, human leukocyte antigen-DR alpha; PDRN, polydeoxyribonucleotide; cAMP, cyclic adenosine monophosphate; HIF-1α, hypoxia-inducible factor-1 alpha; DSS, dextran sulfate sodium; IEC, intestinal epithelial cell; TRAF6, tumor necrosis factor receptor-associated factor 6; MSC, mesenchymal stem cell; Ce-TPP, cerium-based nanozyme functionalized with triphenylphosphonium; ZO-1, zonula occludens-1; TAT, transactivator of transcription; GFP, green fluorescent protein.

**Figure 3:**
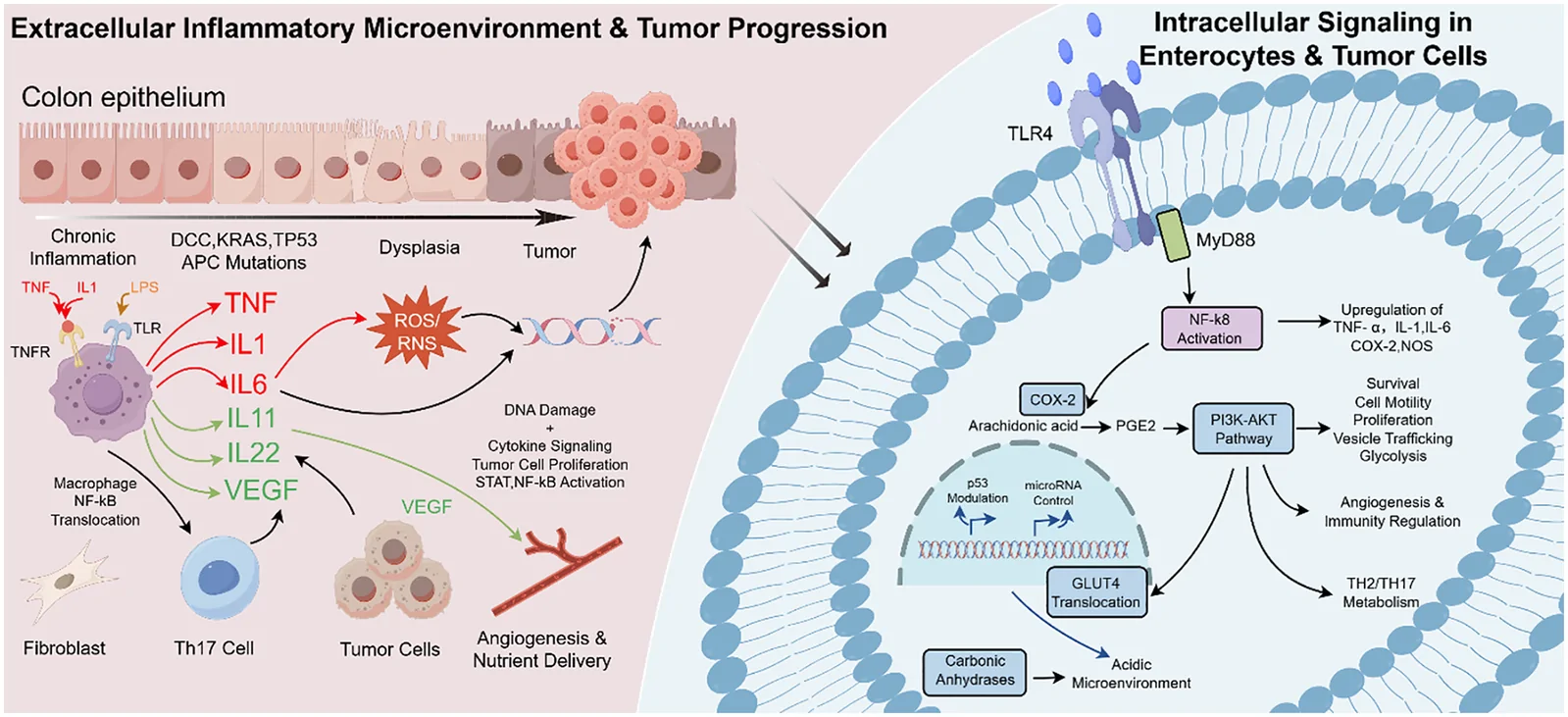
Molecular mechanisms linking chronic intestinal inflammation to CRC in IBD. Chronic inflammation promotes IBD-associated CRC through persistent cytokine activation, oxidative stress, and oncogenic signaling. In the inflammatory microenvironment (left), activated immune cells, including macrophages and Th17 cells, release cytokines such as TNF-α, IL-1β, IL-6, IL-11, IL-22, and VEGF, inducing ROS/RNS production, DNA damage, angiogenesis, and epithelial dysplasia. These processes contribute to the accumulation of mutations in key genes (e.g., APC, KRAS, TP53, and DCC) and malignant transformation. In epithelial and tumor cells (right), inflammatory signals activate TLR4/MyD88-mediated NF-κB, COX-2/PGE2, PI3K/AKT, and STAT3 pathways, promoting proliferation, survival, migration, angiogenesis, EMT, metabolic reprogramming, and immune evasion, thereby driving CRC progression.

## 5 Therapeutic applications of engineered EVs in IBD-associated CRC

Chronic intestinal inflammation is a major driver of CAC, in which persistent epithelial injury, oxidative stress, microbiota dysbiosis, immune dysregulation, and inflammatory signaling contribute to the transition from chronic colitis to dysplasia and carcinoma (140). Accordingly, therapeutic strategies for CAC should ideally target both malignant cells and the inflammatory tumor-promoting environment that links IBD to carcinogenesis. Engineered EVs provide a potentially suitable platform because they can deliver chemotherapeutic agents, nucleic acids, immunomodulators, and other therapeutic cargos while simultaneously influencing inflammatory and tumor-associated pathways (141–143). However, an important distinction should be made between direct evidence in CAC models and evidence extrapolated from conventional CRC models. A substantial proportion of the current literature on engineered EV-based anticancer therapy has been generated using established CRC cell lines, xenograft models, or orthotopic tumor models without pre-existing chronic intestinal inflammation (144, 145). These studies provide valuable proof-of-concept evidence for tumor targeting, cargo delivery, immune modulation, and inhibition of malignant phenotypes, but they do not fully reproduce the pathophysiological features of CAC. In particular, conventional CRC models generally lack the persistent inflammatory injury, dysregulated intestinal barrier, microbiota alterations, and inflammation-dependent immune and stromal interactions that characterize IBD-associated carcinogenesis (146, 147). Therefore, findings from conventional CRC models should be interpreted as supportive or potentially translatable evidence rather than direct evidence of efficacy against CAC. Future studies should prioritize inflammation-associated models, including experimental systems that combine chronic colitis with tumor initiation and progression, to determine whether engineered EVs retain their targeting efficiency, therapeutic activity, and safety within the distinct CAC microenvironment (148).

### 5.1 Targeted delivery of anticancer drugs and gene therapeutics

#### 5.1.1 Precision delivery of chemotherapeutic and genetic cargos to CRC cells

Most available evidence supporting engineered EV-mediated delivery of anticancer drugs and genetic cargos in CRC has been generated using conventional CRC models rather than inflammation-associated CRC or CAC models. For example, engineered extracellular vesicles co-loaded with 5-fluorouracil (5-FU) and miR-21 inhibitors enhanced tumor-suppressive activity and reversed chemoresistance in CRC models (142). Similarly, blood-derived extracellular vesicles carrying doxorubicin and a miR-21 inhibitor produced greater tumor suppression than free drug administration (149). MSC-derived extracellular vesicles modified with targeting molecules, including internalizing-RGD (iRGD) peptides and mucin 1 (MUC1) aptamers, have also demonstrated enhanced accumulation in CRC cells, supporting the potential of surface-engineered EVs for more selective therapeutic delivery (150). Although these studies provide important proof-of-concept evidence for EV-mediated drug targeting, combination delivery, and tumor-cell specificity, the available findings should be distinguished from direct evidence in CAC. Most of these approaches were evaluated in conventional CRC cell lines or tumor models that do not fully reproduce the chronic inflammatory environment associated with IBD-driven carcinogenesis. Shared malignant processes, including tumor proliferation, chemoresistance, invasion, and altered drug responsiveness support their potential relevance to CAC. However, chronic intestinal inflammation may substantially influence EV biodistribution, tumor uptake, cellular targeting, therapeutic efficacy, and toxicity. Therefore, these findings should currently be considered supportive evidence for potential application in CAC rather than direct evidence of efficacy against inflammation-associated CRC. Validation in appropriate colitis-associated tumor models will be necessary to determine whether these engineered EV platforms retain their targeting efficiency and therapeutic activity within the inflammatory CAC microenvironment.

#### 5.1.2 Co-delivery of multiple cargos for synergistic anticancer effects

Tumor heterogeneity and adaptive resistance represent major obstacles to effective CRC treatment. Engineered EVs provide a unique platform for the simultaneous delivery of multiple therapeutic cargos, enabling coordinated regulation of independent yet interconnected cancer pathways. By combining chemotherapeutic drugs with nucleic acid therapeutics, EVs can simultaneously interfere with tumor proliferation, DNA repair, apoptosis resistance, and metastatic signaling pathways, potentially generating stronger therapeutic effects than single-agent approaches (151). For instance, bone marrow mesenchymal stem cell-derived exosomes loaded with miR-16-5p suppress CRC cell proliferation, migration, and invasion by targeting integrin α2 (ITGA2), while promoting apoptosis (152). Similarly, bone marrow-derived mesenchymal stem cell (BMSC)-derived extracellular vesicles delivering miR-4461 inhibit malignant phenotypes in human colorectal carcinoma cell line(HCT116) and human colon adenocarcinoma SW480 cell line(SW480) colorectal cancer cells through suppression of coatomer protein complex subunit beta-2(COPB2), thereby limiting tumor progression (153). These findings illustrate the potential of engineered EVs to function as programmable delivery platforms capable of simultaneously modulating multiple tumor-associated pathways. However, these findings should be distinguished from direct evidence in CAC. The reported effects of miR-16-5p- and miR-4461-loaded BMSC-derived extracellular vesicles were primarily demonstrated in established CRC cell models rather than inflammation-associated CAC models (152, 153). Therefore, their relevance to CAC remains inferential rather than directly demonstrated. Although the targeted pathways are involved in malignant proliferation, invasion, and tumor progression and may therefore be relevant to CAC, conventional CRC models do not fully reproduce the chronic inflammatory environment, altered epithelial barrier function, immune-cell infiltration, and microbiota dysbiosis that characterize IBD-associated carcinogenesis. These disease-specific factors may influence EV uptake, biodistribution, cargo activity, and therapeutic responses. Consequently, whether these multifunctional EV strategies retain comparable efficacy in the inflammatory CAC microenvironment requires direct validation in appropriate colitis-associated tumor models.

### 5.2 EV-Based immunotherapy strategies

#### 5.2.1 Delivery of immune checkpoint inhibitors and immune-stimulatory molecules

Immune evasion is an important feature of CRC progression, and chronic inflammation may further shape the immunosuppressive microenvironment characteristic of CAC. Engineered EVs have emerged as potential delivery systems for immune checkpoint modulators by enabling localized immune activation while potentially reducing systemic immune-related toxicity. Through surface modification or intraluminal cargo loading, EVs can deliver molecules that target immune checkpoint pathways, including programmed cell death-1(PD-1)/PD-L1 inhibitors, immunoregulatory proteins, and nucleic acid therapeutics (154). Compared with systemic antibody administration, EV-based delivery may increase therapeutic retention within the tumor microenvironment and prolong local immune modulation, offering potential advantages for immunologically suppressed CRC and potentially CAC (155). However, most of the current evidence supporting engineered EV-based immunotherapy has been generated in conventional CRC models rather than in inflammation-associated tumor models. Although these studies provide an important mechanistic rationale for applying EV-based immune modulation to CAC, the immune landscape of CAC is shaped by persistent intestinal inflammation, altered myeloid-cell populations, cytokine signaling, and microbiota-derived signals. Consequently, therapeutic efficacy demonstrated in conventional CRC models should not be assumed to directly translate to CAC and requires validation in appropriate inflammation-associated tumor models. In addition to serving as therapeutic carriers, EVs themselves participate in tumor immune regulation. Naturally circulating small EVs carrying immune checkpoint molecules, including PD-1, CD80, and PD-L1, have been implicated in tumor immune escape by regulating checkpoint signaling and immune tolerance (156). These observations suggest that engineered EV-based strategies may have dual potential as therapeutic delivery systems and as approaches for disrupting EV-mediated immunosuppression. Nevertheless, whether these EV-associated immune checkpoint mechanisms have equivalent functional importance in CAC remains insufficiently defined. Furthermore, combining EV delivery with innate immune activation represents another promising strategy. Engineered EVs carrying cyclic GMP-AMP synthase(cGAS), Stimulator of interferon genes (STING) agonists, or other immune-stimulatory molecules may reshape the tumor immune microenvironment and enhance coordination between innate and adaptive immune responses, potentially improving responses to immune checkpoint blockade (157). Such approaches may be particularly relevant to CAC because persistent inflammatory signaling and immune dysregulation are integral components of inflammation-associated carcinogenesis. However, their therapeutic efficacy and safety in the context of chronic intestinal inflammation remain to be established. Future studies using inflammation-associated CRC models should therefore determine whether EV-mediated immune activation can achieve effective tumor control without exacerbating the underlying intestinal inflammatory response.

#### 5.2.2 Antigen delivery for induction of tumor-specific immunity

Beyond immune checkpoint regulation, engineered EVs can function as antigen delivery platforms to stimulate tumor-specific immune responses. EV-mediated antigen presentation can enhance uptake by APCs, promote major histocompatibility complex (MHC)-dependent antigen presentation, and activate tumor-reactive T cells. Red blood cell-derived EVs loaded with ovalbumin (OVA) and modified with anti-DEC-205 targeting molecules significantly enhanced dendritic cell uptake, MHC-I antigen presentation, and subsequent T-cell activation, demonstrating the vaccine potential of engineered EV platforms (158). Similarly, engineered EVs carrying Severe Acute Respiratory Syndrome (SARS-CoV-2)-CoV-2 Spike antigen effectively activated CD4^+^ and CD8^+^ T cells through APC-mediated processing, further supporting the feasibility of EV-based antigen delivery strategies (159). For CRC, antigen delivery strategies may provide additional benefits when combined with tumor microenvironment modulation. This is particularly important in IBD-associated CRC, where chronic inflammation, microbiota alteration, and immune escape continuously interact to promote tumor progression. Recent studies show that tumor-derived engineered EVs carrying miR-206 can regulate both immune responses and gut microbiota composition, suggesting that EV platforms may simultaneously activate anti-tumor immunity and remodel tumor-associated ecosystems (160).

### 5.3 Inhibition of angiogenesis and metastatic progression

Tumor progression and metastasis are strongly influenced by EV-mediated communication between cancer cells, stromal cells, endothelial cells, and immune populations. CRC-derived EVs contribute to angiogenesis, vascular permeability, epithelial–mesenchymal transition (EMT), extracellular matrix remodeling, and the formation of pre-metastatic niches (PMNs) (161). Studies have demonstrated that tumor-derived small EVs promote metastatic progression by activating stromal cells and modifying distant tissue environments. For example, integrin expression on EV surfaces determines organ-specific metastatic patterns, with α6β4 and α6β1 integrins associated with lung metastasis, whereas αvβ5 is linked with liver metastasis (162). Cancer-associated fibroblast (CAF)-derived extracellular vesicles promote angiogenesis through miR-135b-5p-mediated suppression of thioredoxin-interacting protein(TXNIP), while HCT116-derived exosomal miR-1229 enhances endothelial pro-angiogenic signaling, demonstrating the importance of EV-mediated regulation of tumor vascularization (163). Based on these mechanisms, engineered EVs can be designed to deliver anti-angiogenic molecules, inhibitory miRNAs, siRNAs, or therapeutic proteins targeting EMT and extracellular matrix remodeling pathways. Such approaches may simultaneously suppress angiogenesis, invasion, and metastatic dissemination (164). Furthermore, EV surface modification with tumor-targeting peptides, including cRGD and iRGD, enhances accumulation within tumor vasculature and malignant tissues. When combined with chemotherapy agents or nucleic acid therapeutics, these engineered EVs improve therapeutic precision while reducing systemic exposure (165). For IBD-associated CRC, where chronic inflammation, hypoxia, and vascular remodeling create a highly metastatic environment, EV-based interventions targeting angiogenesis and polymorphonuclear leukocyte(PMN) formation may provide a rational strategy for interrupting inflammation-driven cancer progression.

### 5.4 Combination therapy and translational potential of engineered EV platforms

Due to their biocompatibility, programmable engineering capacity, and ability to deliver multiple therapeutic cargos, engineered EVs represent promising partners for combination therapies involving chemotherapy, radiotherapy, and immunotherapy. EV-based combination approaches improve therapeutic efficacy by enhancing tumor accumulation, increasing cellular uptake, enabling controlled release, and remodeling immunosuppressive tumor environments (166, 167). For example, extracellular vesicles carrying PD-L1 siRNA and cytotoxic T-lymphocyte-associated antigen-4 (CTLA-4) siRNA suppress CRC cell proliferation, reduce immune escape, and enhance CD8^+^ T-cell-mediated anti-tumor activity (168). In another study, Lys-Asp-Glu-Leu(KDEL) peptide-modified milk-derived EVs co-delivering celastrol and PD-L1 siRNA promoted immunogenic cell death, increased dendritic cell activation, and enhanced T-cell infiltration, resulting in improved therapeutic efficacy in orthotopic CRC models (169). Because tumor-derived exosomal PD-L1 contributes to local and systemic immunosuppression, targeting EV production, secretion, or uptake pathways may further enhance sensitivity to PD-1/PD-L1 blockade therapies (170).

Despite these promising findings, clinical translation of engineered EV-based therapies remains limited by several challenges, including manufacturing standardization, batch-to-batch consistency, cargo-loading efficiency, biodistribution control, pharmacokinetic characterization, and large-scale production (171, 172). Addressing these challenges will be essential for transforming engineered EV platforms from experimental approaches into clinically applicable therapies. Importantly, the current evidence base for engineered EV therapy in IBD-associated CRC remains limited by the scarcity of studies performed in experimentally defined CAC models (173–175). Many reported studies rely on CRC cell lines, subcutaneous xenografts, or orthotopic tumor models that reproduce malignant growth but do not fully recapitulate the chronic inflammatory environment underlying CAC development (176, 177). This distinction is particularly relevant because CAC differs from sporadic CRC in its initiating mechanisms, molecular evolution, immune landscape, stromal interactions, and relationship with the intestinal microbiota (177, 178). Consequently, an engineered EV platform that demonstrates efficient tumor targeting or antitumor activity in a conventional CRC model may not necessarily exhibit the same biodistribution, cellular uptake, therapeutic efficacy, or safety in inflammation-associated tumors. Conversely, EVs designed to modulate inflammatory pathways may exert additional effects in CAC that are not captured by conventional CRC models (179, 180). Future studies should therefore prioritize clinically relevant inflammation-associated models, particularly chronic colitis–tumor models, and evaluate both tumor control and modulation of the inflammatory microenvironment. Direct comparison of engineered EV efficacy between conventional CRC and CAC models will be important for determining which therapeutic mechanisms are conserved and which are specific to inflammation-driven carcinogenesis. Representative engineered EV-based therapeutic approaches for IBD-associated CRC, including targeted drug delivery, immune modulation, metastasis inhibition, and combination therapy, are summarized in **Table 3**.

**Table 3:** Representative engineered extracellular vesicle-based therapeutic strategies with current or potential application to colitis-associated colorectal cancer (CAC).

EV source	Engineering strategy	Therapeutic cargo/modification	Experimental model	Major therapeutic outcome	Relevance to CAC	Level of evidence	Refs.
Engineered EVs	Targeted drug/gene delivery	Chemotherapeutic drugs or nucleic acids	CRC cell lines/xenografts	Enhanced tumor targeting and reduced systemic toxicity	Potential strategy for CAC treatment	Preclinical	(239, 240)
Engineered extracellular vesicles	Co-delivery	5-FU + miR-21 inhibitor	CRC xenograft	Reversed chemoresistance through restoration of hMSH2/PTEN signaling	Potential application in CAC	Mouse	(142)
Blood-derived extracellular vesicles	Drug/miRNA co-loading	Doxorubicin + miR-21 inhibitor	CRC xenograft	Enhanced tumor suppression with reduced off-target toxicity	Potential application in CAC	Mouse	(149)
MSC-derived extracellular vesicles	Surface modification	iRGD + MUC1 aptamer	CRC models	Improved tumor homing and targeted drug delivery	Potential targeted therapy for CAC	Preclinical	(150)
BMSC-derived extracellular vesicles	miRNA loading	miR-16-5p	CRC cells/xenograft	Suppressed ITGA2-mediated proliferation and metastasis	Potential molecular therapy for CAC	Preclinical	(152)
BMSC-derived extracellular vesicles	miRNA loading	miR-4461	CRC models	Inhibited COPB2 and malignant progression	Potential molecular therapy for CAC	Preclinical	(153)
Engineered EVs	Immune checkpoint delivery	PD-1/PD-L1 inhibitors	Tumor models	Enhanced anti-tumor immunity	May improve immunotherapy for advanced CAC	Preclinical	(154–157)
Erythrocyte-derived EVs	Antigen engineering	OVA + anti-DEC-205	Tumor models	Enhanced dendritic cell activation and T-cell responses	Potential therapeutic vaccine for CAC	Preclinical	(158, 159)
Engineered EVs	Anti-angiogenic targeting	Anti-angiogenic cargos; cRGD/iRGD modification	CRC models	Suppressed angiogenesis and metastasis	May limit CAC progression	Preclinical	(161–165)
Engineered EVs	Combination therapy	Chemotherapy, PD-L1 siRNA, CTLA-4 siRNA, celastrol	CRC models	Enhanced immune activation and therapeutic efficacy	Potential multimodal strategy for CAC	Preclinical	(166–170)

EV, extracellular vesicle; CRC, colorectal cancer; CAC, colitis-associated colorectal cancer; 5-FU, 5-fluorouracil; miR, microRNA; MSC, mesenchymal stem cell; BMSC, bone marrow-derived mesenchymal stem cell; iRGD, internalizing arginine-glycine-aspartic acid peptide; MUC1, mucin 1; hMSH2, human MutS homolog 2; PTEN, phosphatase and tensin homolog; PD-1, programmed cell death protein 1; PD-L1, programmed death-ligand 1; OVA, ovalbumin; DEC-205, dendritic cell receptor DEC-205; cRGD, cyclic arginine-glycine-aspartic acid peptide; siRNA, small interfering RNA; CTLA-4, cytotoxic T-lymphocyte-associated protein 4.

## 6 Sources of EVs for engineering and therapeutic applications

The biological source of EVs is a critical determinant of their therapeutic performance, influencing cargo composition, targeting capacity, immunogenicity, scalability, and clinical applicability. For conceptual clarity, vesicular nanoparticles used in biomedical studies should be classified according to their biological origin and, where possible, their biogenesis. The first category comprises mammalian cell-derived EVs. This group includes EVs released by mesenchymal stromal cells, immune cells, epithelial cells, and tumor cells. The second category is naturally derived EVs, including plant-derived vesicles (PDVs), milk-derived EVs (MEVs),and bacterial extracellular vesicles (BEVs). PDVs are particles isolated from homogenized plant tissues or juices, and for these particles, endogenous EV biogenesis cannot be definitively established. MEVs are topically isolated from mammalian biofluids such as milk. BEVs, also referred to as bacterial membrane vesicles, include outer membrane vesicles derived from Gram-negative bacteria and cytoplasmic membrane vesicles from Gram-positive bacteria. Cell-derived EVs generally exhibit superior biological compatibility and endogenous targeting characteristics, whereas naturally derived EVs offer advantages in accessibility, stability, and large-scale production. Therefore, selecting an appropriate EV source is essential for developing effective engineered EV platforms for inflammatory diseases and cancer, particularly in the context of IBD and IBD-associated CRC.

### 6.1 Cell-derived EVs

#### 6.1.1 Mesenchymal stem cell-derived EVs

MSCs are multipotent stromal cells with self-renewal capacity, immunomodulatory properties, and regenerative potential, making them among the most widely studied cellular sources for therapeutic EV production (181). The therapeutic functions of MSCs are largely mediated through paracrine mechanisms, including secretion of soluble factors and release of extracellular vesicles containing functional proteins, mRNAs, and microRNAs (182). Importantly, accumulating evidence suggests that MSC-derived EVs can recapitulate many of the beneficial effects of parental MSCs while avoiding several limitations associated with cell transplantation, including uncontrolled differentiation, poor survival after transplantation, and potential safety concerns (51). Therefore, MSC-derived EVs have emerged as promising cell-free alternatives for regenerative medicine and immune-related disorders. MSCs from different tissue origins, including human umbilical cord-derived MSCs (hUC-MSCs), bone marrow-derived MSCs (hBM-MSCs), adipose-derived MSCs (hAD-MSCs), and olfactory ecto-mesenchymal stem cells (OE-MSCs), produce EVs with distinct molecular compositions and therapeutic properties. Their favorable immunomodulatory profile, cargo flexibility, and tissue repair capacity make MSC-EVs particularly attractive for engineering applications in IBD and CRC. However, challenges, including donor variability, EV heterogeneity, production scalability, and manufacturing standardization, remain significant barriers to clinical translation (183–185).

#### 6.1.2 Immune cell-derived EVs: dendritic cell-derived EVs

Dendritic cells (DCs) are professional antigen-presenting cells that regulate antigen recognition, immune activation, and immune tolerance. EVs derived from DCs (DEVs) retain immune-related molecules, including peptide-major histocompatibility complex (MHC) complexes and co-stimulatory molecules, thereby enabling communication with immune cells and regulating adaptive immune responses (186). Early studies demonstrated the feasibility of using immune cell-derived EVs as therapeutic delivery systems. Alvarez-Erviti et al. showed that rabies virus glycoprotein peptide(RVG)-modified extracellular vesicles could deliver RNA interference molecules across the blood–brain barrier, providing important evidence for targeted EV-based delivery (55). Subsequently, El-Andaloussi et al. established comprehensive workflows for EV isolation, characterization, siRNA loading, and functional delivery, accelerating the development of engineered EV technologies (187). Because DEVs combine natural immune activation properties with favorable biocompatibility and relatively low immunogenicity, they have been extensively explored for cancer immunotherapy, vaccine development, and immune modulation. Nevertheless, their clinical translation is limited by challenges related to large-scale production, quality control, and manufacturing complexity (188).

#### 6.1.3 Intestinal epithelial cell-derived EVs

IECs are a biologically relevant source of EVs in gastrointestinal diseases because they directly contribute to the maintenance of intestinal homeostasis. EVs isolated from colonic lavage fluid express epithelial markers such as epithelial cell adhesion molecule (EpCAM), together with classical exosomal markers including CD63 and CD9, confirming their epithelial origin. IEC-derived EVs have attracted attention as gastrointestinal-compatible delivery vehicles. Compared with free curcumin, curcumin-loaded IEC-EVs significantly improved cellular uptake and intestinal permeability in Caco-2 models, suggesting their potential for oral therapeutic delivery (189). Furthermore, IEC-derived EVs contain functional microRNAs that regulate inflammatory responses and maintain epithelial homeostasis (190). In experimental neonatal necrotizing enterocolitis models, IEC-EVs improved survival, reduced intestinal injury, restored epithelial architecture, promoted angiogenesis, and decreased inflammatory signaling, highlighting their regenerative potential (191). However, compared with MSC-EVs, IEC-derived EVs remain less extensively characterized. Further studies are required to clarify their molecular mechanisms, isolation procedures, therapeutic consistency, and long-term safety.

#### 6.1.4 Tumor cell-derived EVs

Tumor-derived EVs (TEVs) possess unique biological characteristics because they inherit molecular components from their parental tumor cells, including membrane proteins, lipids, tumor-associated antigens, and nucleic acids. These characteristics provide TEVs with intrinsic homotypic targeting properties, allowing preferential interaction with tumor cells. For example, HT1080-derived EVs exhibited greater accumulation in the corresponding tumor tissues than HeLa-derived EVs, suggesting that EV targeting efficiency is strongly influenced by parental cell origin (192). In addition, TEVs contain tumor-associated antigens and immune-related molecules that can stimulate T-cell-mediated anti-tumor responses (193). Preclinical studies have demonstrated the therapeutic potential of TEVs in melanoma, hepatocellular carcinoma, and colorectal cancer models through tumor-specific recognition and immune activation mechanisms (194–196). However, their clinical application remains challenging due to tumor heterogeneity, potential transfer of oncogenic molecules, and complex interactions with the tumor immune microenvironment.

### 6.2 Naturally derived EVs

#### 6.2.1 Plant-derived EVs

Plant-derived vesicles (PDVs) have emerged as attractive EV platforms because of their abundance, low production cost, biocompatibility, and potential for oral administration. Although plant vesicles were first described in 1967, their therapeutic applications have only recently gained attention (197). Unlike mammalian EVs, PDVs offer several advantages, including sustainable production, low toxicity, and resistance to gastrointestinal degradation. Furthermore, surface modification strategies can improve PDV targeting efficiency and therapeutic performance (198). Edible plant-derived EVs from ginger, grape, grapefruit, carrot, and turmeric have demonstrated anti-inflammatory, antioxidant, immunomodulatory, and anticancer activities (199, 200). For example, turmeric-derived EVs alleviate colitis by regulating heme oxygenase-1 (HO-1) antioxidant pathways and inflammatory cytokine expression, whereas ginger-derived EVs suppress cyclin D1 expression in colorectal cancer models (201, 202). Despite their advantages, limitations remain regarding PDV biogenesis mechanisms, functional characterization, isolation standardization, and purification efficiency, which currently restrict clinical application (203, 204).

#### 6.2.2 Milk-derived EVs

Milk-derived EVs (MEVs) are among the most promising naturally occurring EV platforms due to their abundance, stability, and suitability for oral delivery. Milk EVs contain proteins, lipids, and nucleic acids and remain stable under harsh gastrointestinal conditions, including acidic environments (205). MEVs can encapsulate both hydrophilic and hydrophobic compounds, making them suitable carriers for therapeutic drugs. Studies have shown that milk EVs improve the delivery and anti-tumor activity of chemotherapeutic agents compared with free drugs (206). Moreover, MEVs can be absorbed via neonatal Fc receptor (FcRn)-mediated pathways, thereby enhancing the stability, solubility, and bioavailability of therapeutic molecules such as curcumin (207, 208). In addition, milk-derived EVs can influence intestinal epithelial proliferation, microbiota composition, and immune regulation (209, 210). Although MEVs have considerable translational potential, further studies are required to evaluate long-term safety, batch consistency, and potential immunological effects associated with repeated administration.

#### 6.2.3 Bacterial EVs

Bacterial extracellular vesicles (BEVs) are lipid-bound nanoparticles released by both Gram-positive and Gram-negative bacteria. They mediate communication between bacteria and host cells by transferring proteins, nucleic acids, lipids, and other bioactive molecules (211). BEVs can influence host immune responses, bacterial pathogenicity, and antibiotic resistance mechanisms. Their molecular composition reflects bacterial physiological states, making them potential diagnostic biomarkers for infectious diseases, inflammatory disorders, and cancer (212–214). Due to their immunogenic properties, BEVs have also been investigated as vaccine platforms. A vaccine strategy combining the SARS-CoV-2 spike receptor-binding domain with Salmonella typhimurium-derived EVs successfully induced neutralizing antibody responses against viral variants, demonstrating the EVs’ potential for antigen delivery (215). BEVs have been further explored as oral antimicrobial delivery systems. Antibiotic-loaded BEVs showed prolonged intestinal retention, enhanced antibacterial activity, and reduced disruption of the gut microbiota compared with free antibiotics in infection models (216). However, clinical translation of BEVs remains limited by concerns about immunotoxicity, standardization of purification, and reproducibility in manufacturing. Future studies must establish reliable strategies for production and characterization to facilitate their therapeutic application (217, 218).

## 7 Challenges and future perspectives

Despite remarkable advances in EV engineering, several scientific, technological, and regulatory challenges continue to impede the clinical translation of engineered EVs for the treatment of IBD and IBD-associated CRC. One of the primary obstacles is the lack of standardized methods for EV isolation, purification, and large-scale manufacturing. Current techniques, including ultracentrifugation, ultrafiltration, immunoaffinity capture, and microfluidic-based approaches, each involve trade-offs between purity, yield, processing time, and scalability, making it difficult to establish reproducible Good Manufacturing Practice (GMP)-compliant manufacturing processes (115). Moreover, the intrinsic heterogeneity and relatively low production yields of EVs remain major barriers to the consistent production of therapeutic products (116). Another critical challenge is the need for comprehensive EV characterization and quality control. Standardized evaluation should extend beyond particle size and concentration to include cargo profiling, biological potency assays, sterility testing, and batch-to-batch consistency. Such quality-control measures are particularly important for EVs derived from diverse sources, including mammalian cells, plants, and bacteria, which exhibit distinct molecular compositions and biological properties (219). Establishing internationally accepted standards for EV characterization and release criteria will be essential for regulatory approval and clinical application. Improving the *in vivo* performance of engineered EVs also remains an important research priority. Although EVs possess excellent biocompatibility and intrinsic targeting capabilities, enhancing tissue-specific accumulation while minimizing off-target biodistribution is necessary to maximize therapeutic efficacy. Surface functionalization with targeting ligands, biomimetic membrane engineering, and stimuli-responsive modifications may improve selective delivery to inflamed intestinal tissues or colorectal tumors. In addition, maintaining EV stability during storage, transportation, and administration remains challenging, thereby encouraging the development of protective formulations, such as hydrogels and other controlled-release systems, to preserve biological activity and prolong therapeutic efficacy (220).

Looking ahead, personalized EV-based therapy represents a potential direction for precision treatment of IBD and IBD-associated CRC (172, 221). Rather than using a uniform EV formulation for all patients, the EV source, therapeutic cargo, targeting ligand, and delivery route could be selected based on patient-specific inflammatory, epithelial, microbial, and tumor characteristics. For example, patients with predominant TNF-α-, IL-6-, or NF-κB-associated inflammatory activity could be stratified for EVs enriched with anti-inflammatory cargo designed to attenuate excessive immune activation (222). In contrast, patients with prominent epithelial barrier dysfunction could be considered candidates for EVs engineered to target intestinal epithelial cells and deliver barrier-repair cargo preferentially (223). In IBD-associated CRC, tumor-associated molecular or immune characteristics could potentially guide the selection of tumor-targeting ligands or cargos directed against oncogenic signaling and immunosuppressive pathways (224). Similarly, expression of patient-specific tumor-associated surface markers could potentially be used to select EV modifications that enhance tumor-cell recognition while limiting uptake by non-target tissues (144, 225).

The gut microbiome may provide an additional layer for patient stratification and EV design. Differences in microbial community composition and microbial metabolites associated with persistent inflammation, epithelial dysfunction, or inflammation-driven carcinogenesis could potentially identify patients for whom microbiota-directed EV strategies are more appropriate (226). For instance, patients with pronounced dysbiosis or enrichment of inflammation-associated microbial taxa could potentially be considered for orally administered or microbiota-targeted EV formulations designed to modulate host–microbiota interactions (227). Longitudinal changes in microbial composition or metabolite profiles could also serve as pharmacodynamic indicators of treatment response, provided that their relationships with EV exposure and clinical outcomes are established (226). Thus, patient-specific biomarkers could potentially be used not only to identify treatment candidates but also to inform the selection of EV cargo, targeting strategy, and delivery route. However, this biomarker-guided framework remains largely prospective. The available evidence does not yet establish validated biomarker thresholds for selecting specific EV cargos or engineering strategies, nor has it demonstrated that microbiome profiles can reliably predict responses to engineered EV therapy (172). Future studies should therefore integrate molecular, immune, epithelial, and microbiome profiling with EV pharmacokinetic and pharmacodynamic analyses to determine whether patient-specific characteristics can meaningfully guide EV design and treatment selection. Biomarker-stratified preclinical studies and subsequently designed clinical trials will be necessary to determine whether personalized EV engineering can improve therapeutic efficacy, reduce off-target effects, and support precision treatment across the IBD-to-CAC continuum (228). Furthermore, combining engineered EVs with biologics, chemotherapy, immunotherapy, or gene-editing technologies may yield synergistic therapeutic effects and overcome drug resistance. Achieving successful clinical translation will require scalable GMP-compliant manufacturing, standardized separation and characterization protocols, rigorous potency and efficacy testing, and well-designed clinical trials with harmonized endpoints, including mucosal healing, tumor response, long-term safety, biodistribution, repeated-dose tolerability, and immunogenicity (116, 219).

### 7.1 Current clinical development and translational consideration

Many preclinical studies described as “engineered EV therapies” incorporate modifications to enhance cargo loading, colonic localization, epithelial uptake, immune-cell targeting, or tumor selectivity. However, clinical translation remains limited, as existing IBD studies have primarily evaluated non-engineered EV-containing products, and no registered interventional clinical trials have yet directly assessed rigorously engineered EV products incorporating intentional cargo loading, surface ligand modification, or genetic engineering for IBD or CRC (229–231). Engineered EV-based approaches for CRC, including the delivery of chemotherapeutic agents, siRNA/miRNA, CRISPR-associated cargoes, immune agonists, and tumor-targeting ligands, remain largely at the preclinical stage and should not yet be considered clinically established therapies. Their successful translation will require standardized GMP manufacturing, validated multiparametric potency assays, reproducible storage and distribution conditions, and comprehensive biodistribution and repeated-dose safety evaluations. Regulatory requirements may also vary across jurisdictions. Importantly, clinical development requires EVs to be characterized and manufactured as defined biological drug products rather than as poorly characterized conditioned media or generic “exosome” preparations. Manufacturing scale-up should ensure consistent batch-to-batch quality, including vesicle identity, purity, structural integrity, cargo composition, and biological activity. Recent development of GMP-compatible processes for EV-enriched secretomes derived from cardiovascular progenitor cells demonstrates the feasibility of implementing appropriate process and release controls. Nevertheless, these manufacturing frameworks will require further optimization and indication-specific validation for applications in intestinal inflammation and CRC (232, 233). Because cargo engineering can generate multiple biological effects, a panel of complementary potency assays is likely to provide more robust evidence than reliance on a single functional readout. For IBD-focused EV products, relevant assays may assess the suppression of macrophage-derived TNF and IL-6, inhibition of NF-κB or STAT3 activation, restoration of transepithelial electrical resistance, reduction of epithelial permeability, promotion of epithelial wound repair, and regulation of T-cell polarization (62). For CAC/CRC applications, potency evaluation should additionally confirm target-cell uptake, effective delivery and biological activity of the engineered cargo, inhibition of tumor proliferation or induction of apoptosis, activation of antitumor immune responses, and modulation of the immunosuppressive tumor microenvironment (234). In terms of storage and distribution, freezing at approximately −80 °C is commonly used during early-stage development. However, repeated freeze–thaw cycles can induce vesicle aggregation, membrane damage, cargo leakage, and reduced cellular uptake or biological activity. Accordingly, stability studies should evaluate particle concentration and size, aggregation, membrane integrity, cargo retention, biological potency, sterility, and container compatibility under both real-time and accelerated storage conditions (235). Biodistribution is a critical consideration for EV-based therapies targeting IBD and CAC, as intravenously administered EVs are commonly distributed to the liver, spleen, lungs, and mononuclear phagocyte system. For IBD applications, biodistribution studies should assess EV accumulation within the colonic mucosa, distinguish uptake by epithelial and immune cells, determine retention at inflamed sites, and evaluate systemic exposure following local administration, while considering the potential influence of microbiota composition, mucus disruption, and increased intestinal permeability (62, 234, 235). In CAC/CRC, studies should additionally characterize tumor-to-liver and tumor-to-spleen distribution, intratumoral penetration, differential uptake by malignant, stromal, and immune cells, and the efficiency of engineered cargo release within target cells (57). Repeated dosing will be an important consideration for EV therapies intended for chronic IBD, prevention or interception of CAC in high-risk populations, and combination treatment of CRC. Future preclinical and clinical studies should incorporate dose-escalation and repeated-dose designs, with appropriate monitoring of anti-product antibodies, complement and coagulation responses, hepatic and renal function, inflammatory biomarkers, biodistribution and pharmacokinetics, as well as long-term risks such as malignancy and immune-related adverse events (230, 236). Regulatory classification varies according to the jurisdiction and specific characteristics of the EV product. Early consultation with regulatory authorities is therefore recommended, particularly for engineered EVs incorporating nucleic acids, gene-editing elements, synthetic targeting ligands, or bacterial-vesicle components, which may require additional quality controls, nonclinical safety evaluation, and assessment as combination products. To date, clinical development of EV-based therapies for IBD remains limited to small early-phase studies involving non-engineered bmMSC-EVs or EV-enriched secretome preparations, while EV-based interventions for CAC and CRC have not yet progressed to clinical evaluation. Advancing engineered EV platforms toward clinical application will require GMP-compatible manufacturing, mechanism-based potency assays, validated storage and biodistribution protocols, comprehensive repeat-dose toxicology, and product-specific regulatory consultation to establish whether their proposed benefits in intestinal targeting and cancer interception can be safely and effectively translated to patients.

## 8 Conclusion

Engineered EVs have emerged as a highly promising therapeutic platform for managing both IBD and IBD-associated CRC by integrating targeted drug delivery with the intrinsic biological functions of natural EVs. Beyond serving as nanocarriers, EVs actively participate in regulating immune responses, maintaining intestinal barrier integrity, modulating gut microbiota, and remodeling the tumor microenvironment. Their diverse cargos, including non-coding RNAs, proteins, lipids, and other bioactive molecules, enable simultaneous modulation of multiple signaling pathways involved in chronic intestinal inflammation and inflammation-driven colorectal carcinogenesis (237, 238). Recent advances in EV engineering, including optimized cargo loading, surface functionalization, hybrid biomaterial systems, and the exploration of diverse EV sources, have substantially enhanced targeting specificity, biological stability, and therapeutic efficacy. Strategies such as donor-cell engineering, ligand-mediated targeting, and rational cargo selection have further expanded the potential of EVs for precision treatment by improving selective accumulation within inflamed intestinal mucosa and tumor-associated lesions while minimizing off-target effects (219, 237). Although important challenges remain regarding standardized manufacturing, quality control, targeting efficiency, storage stability, and regulatory approval, continued progress in bioengineering, biomaterials, and precision medicine is expected to accelerate the clinical translation of engineered EVs. In particular, personalized EV strategies may ultimately integrate inflammatory signatures, epithelial and immune phenotypes, microbiome composition, and tumor-associated biomarkers to guide therapeutic cargo selection, targeting ligand design, and delivery route. Such biomarker-guided approaches could enable more precise matching of EV engineering strategies to individual disease characteristics. However, their clinical utility remains to be established through biomarker-stratified preclinical studies and clinical trials. When combined with therapies involving biologics, chemotherapy, or immunotherapy, these advances may provide safer, more effective, and disease-specific treatment options. With continued technological refinement and rigorous clinical validation, engineered EVs have the potential to evolve from experimental nanotherapeutics into clinically applicable precision medicine platforms for both IBD and IBD-associated CRC (238).

## Glossary

IBD: Inflammatory bowel disease
CAC: colitis-associated colorectal cancer
EVs: Extracellular vesicles
UC: ulcerative colitis
CD: Crohn’s disease
EIMs: extraintestinal manifestations
CRC: Colorectal cancer
SIRs: standardized incidence ratios
TNF: tumor necrosis factor
ROS: reactive oxygen species
RNS: reactive nitrogen species
MVs: microvesicles
BBB: blood-brain barrier
mRNAs: messenger RNAs
miRNAs: microRNAs
siRNAs: small interfering RNAs
lncRNAs: long non-coding RNAs
circRNAs: circular RNAs
MSCs: mesenchymal stem cells
IECs: intestinal epithelial cells
TGF-β: transforming growth factor-β
IL-10: interleukin-10
VEGF: vascular endothelial growth factor
EGF: epidermal growth factor
FGF: fibroblast growth factor
LAMP-2B: lysosome-associated membrane protein-2B
cRGD: cyclic arginine-glycine-aspartate
HER2: human epidermal growth factor receptor 2
PD-L1: programmed death-ligand 1
HA: Hyaluronic acid
PDGFR: platelet-derived growth factor receptor
PTGFRN: prostaglandin F2 receptor negative regulator
CXCR4: C-X-C chemokine receptor type 4
TNFR1: tumor necrosis factor receptor 1
IL-6ST: interleukin-6 signal transducer
LPS: lipopolysaccharide
PDRN: polydeoxyribonucleotide
TJ: Tight junction
ZO-1: zonula occludens-1
DCEVs: double-coated extracellular vesicles
TAT: cell-penetrating peptide
GFP: green fluorescent protein
5-FU: 5-fluorouracil
ITGA2: integrin α2
APCs: antigen-presenting cells
OVA: ovalbumin
EMT: epithelial–mesenchymal transition
PMNs: pre-metastatic niches
CAF: Cancer-associated fibroblast
hUC-MSCs: human umbilical cord-derived MSCs
hBM-MSCs: human bone marrow-derived MSCs
hAD-MSCs: human adipose-derived MSCs
OE-MSCs: olfactory ecto-mesenchymal stem cells
DCs: Dendritic cells
DEVs: EVs derived from DCs
MHC: major histocompatibility complex
TEVs: Tumor-derived EVs
PDVs: Plant-derived vesicles
MEVs: Milk-derived EVs
BEVs: Bacterial extracellular vesicles
GMP: Good Manufacturing Practice.
